# Integrating 6G technology in smart hospitals: challenges and opportunities for enhanced healthcare services

**DOI:** 10.3389/fmed.2025.1534551

**Published:** 2025-04-04

**Authors:** Arun Kumar, Mehedi Masud, Mohammed H. Alsharif, Nishant Gaur, Aziz Nanthaamornphong

**Affiliations:** ^1^Department of Electronics and Communication Engineering, New Horizon College of Engineering, Bengaluru, India; ^2^Department of Computer Science, College of Computers and Information Technology, Taif University, Taif, Saudi Arabia; ^3^Department of AI Convergence Electronic Engineering, Sejong University, Seoul, Republic of Korea; ^4^Department of Physics, JECRC University, Jaipur, India; ^5^College of Computing, Prince of Songkla University, Phuket, Thailand

**Keywords:** health care, telemedicine, 6G, smart hospital, AI, IoT, advanced waveforms, big data

## Abstract

**Introduction:**

The advent of sixth-generation (6G) wireless communication technology promises to transform various sectors, with healthcare—particularly smart hospitals—standing to gain significantly. This study investigates the transformative potential of 6G in healthcare by exploring its architectural foundations and enabling technologies.

**Methods:**

A comprehensive review and analysis were conducted on current technological trends, frameworks, and integration strategies relevant to 6G-enabled healthcare systems. The proposed model integrates key technologies such as the Internet of Things (IoT), artificial intelligence (AI), blockchain, robotics, telemedicine, and advanced data analytics within the context of smart hospitals.

**Results:**

The findings suggest that 6G's ultralow latency, massive device connectivity, and high data throughput can dramatically enhance patient care, real-time monitoring, and hospital operational efficiency. The proposed 6G-based smart hospital model fosters seamless communication between medical devices and systems, enabling intelligent decision-making and optimized resource allocation.

**Discussion:**

Despite the promising benefits, several challenges were identified, including data privacy and security risks, system interoperability, and ethical implications. The study underscores the critical importance of robust regulatory frameworks and standardized protocols to ensure secure and ethical deployment of 6G technologies in healthcare settings.

**Conclusion:**

By providing a forward-looking analysis of the opportunities and challenges associated with 6G-powered smart hospitals, this research offers valuable insights into the evolving landscape of digital healthcare and its potential to redefine patient care and hospital management in the near future.

## 1 Introduction

As an evolutionary successor to fifth-generation (5G) technology, 6G represents a significant advancement in wireless communication. It is distinguished by ultrafast data speeds, virtually zero latency, and the capability to support an unprecedented number of connected devices (1). In the context of smart hospitals, the infusion of 6G facilitates real-time communication among a myriad of medical devices, sensors, and systems, laying the foundation for a seamlessly interconnected healthcare ecosystem. This study meticulously predicts the evolution of smart hospitals in the 6G era, shedding light on the intricate network of technologies underpinning this transformative healthcare model. The future of healthcare is entering an era of unprecedented connectivity and technological sophistication, with the imminent arrival of 6G, the sixth generation of wireless communication (2). As we stand on the cusp of this groundbreaking evolution, 6G-based smart healthcare has been poised to revolutionize the medical landscape, offering unparalleled speed, reliability, and transformative capabilities. The core of 6G's potential impact on healthcare is its ability to provide ultrafast data transmission and remarkably low latency (3). These features are critical for enabling real-time communication between medical devices, facilitating the rapid exchange of patient data, and supporting responsive, time-sensitive applications. With the ability to transmit massive amounts of data at lightning speed, 6G sets the stage for a healthcare ecosystem that is not only interconnected, but also operates with unparalleled efficiency. The integration of 6G technology into smart healthcare systems promises to enhance remote patient monitoring, diagnostics, and treatment planning (4). Medical professionals will have access to real-time, high-resolution data, enabling more accurate and timely decision making. This capability is particularly crucial in emergency situations where split-second decisions can significantly affect patient outcomes (5). The effect of 6G on telemedicine was also set to be transformative. Enhanced connectivity facilitates seamless and immersive virtual healthcare experiences, allowing for high-quality video consultation, remote surgery, and interactive patient engagement (6). The geographical barriers that traditionally have limited access to healthcare services will be further dismantled, providing individuals in remote or underserved areas with unprecedented access to medical expertise (7). Furthermore, the integration of 6G with advanced techniques can create a network of interconnected medical devices and wearables, fostering continuous and comprehensive health monitoring. This interconnected ecosystem will contribute to a holistic approach to healthcare, providing a more complete picture of an individual's health and enabling personalized, data-driven interventions. The advent of 6G technology has heralded a new era for smart healthcare, promising to transform the way we access, deliver, and experience medical care (8). The convergence of ultrafast communication, real-time data transmission, and seamless connectivity positions 6G as a catalyst for a healthcare revolution, ushering in an era of unprecedented efficiency, accessibility, and personalized health management. In essence, this comprehensive study embarks on an expedition into the future of healthcare, a future where 6G-based smart hospitals transcend traditional boundaries, ushering in an era of unparalleled connectivity, efficiency, and patient-centric care. Through an examination of architectural evolution, advanced techniques, and challenges, this research seeks to unravel the intricate interplay between technology and healthcare, laying the groundwork for a transformative journey into the era of 6G-enabled smart healthcare ecosystems (9). The integrations of the 6G technology into smart hospitals offers transformative potential by leveraging its ultra-high-speed connectivity, low latency, and massive device connectivity. This integration enables real-time data transmission and processing, facilitating advanced applications such as remote surgeries, AI-driven diagnostics, and enhanced telemedicine services. With 6G, healthcare providers can utilize edge computing to process data locally, reducing latency and ensuring rapid decision-making. The deployment of smart sensors and IoT devices throughout hospital infrastructure allows for continuous patient monitoring, predictive maintenance of medical equipment, and efficient resource management. Furthermore, 6G's enhanced security features ensure the protection of sensitive patient data, mitigating risks associated with cyber threats. The technology also supports seamless communication between various hospital departments, improving operational efficiency and patient care coordination. However, challenges such as the need for significant infrastructure upgrades, high implementation costs, and the requirement for healthcare professionals to adapt to new technologies must be addressed. Despite these challenges, the integration of 6G in smart hospitals presents an opportunity to revolutionize healthcare delivery, offering personalized, efficient, and secure medical services tailored to the needs of individual patients.

The integration of 6G technology in smart hospitals presents significant challenges, primarily due to the need for extensive infrastructure upgrades, high costs, and the complexity of managing vast amounts of data. The deployment of 6G requires a robust network infrastructure capable of supporting ultra-low latency, high data rates, and massive device connectivity. However, existing hospital infrastructure may not be equipped to handle these demands, necessitating substantial investments in new technology, including advanced routers, servers, and edge computing devices. Additionally, the cost of implementing 6G technology can be prohibitive, particularly for smaller or less-resourced healthcare facilities. Another major challenge is the management of the enormous amounts of data generated by 6G-enabled devices, which requires sophisticated data processing and storage solutions to ensure efficient operation.

Advanced techniques like IoT, AI, blockchain, telemedicine, robotics, and advanced data analytics play crucial roles in overcoming the challenges of integrating 6G technology into smart hospitals. IoT enables seamless connectivity between medical devices and systems, ensuring real-time monitoring and data collection from patients, which 6G can then transmit and process at unprecedented speeds. This reduces latency issues and enhances the responsiveness of healthcare services. AI aids in managing the vast amounts of data generated by IoT devices, analyzing patterns for predictive diagnostics, personalized treatment plans, and efficient resource allocation. By automating complex tasks, AI helps alleviate the burden on healthcare professionals, allowing them to focus more on patient care. Blockchain technology addresses security concerns by providing a decentralized and immutable ledger for patient records, ensuring data integrity and privacy. This is particularly important in a 6G-powered environment where data exchange is rapid and extensive. Telemedicine, supported by 6G's low latency, becomes more reliable, enabling high-quality remote consultations and even remote surgeries, expanding access to specialized care regardless of location. Robotics integrated with 6G allows for more precise and real-time control in surgical procedures, improving outcomes while reducing the risk of human error. Finally, advanced data analytics enables hospitals to process and interpret large datasets quickly, offering insights that can lead to improved patient outcomes and operational efficiency. By leveraging these advanced technologies, the challenges of implementing 6G in smart hospitals—such as infrastructure demands, high costs, and the complexity of managing vast amounts of data—can be effectively mitigated, paving the way for a more connected and intelligent healthcare system.

### 1.1 Motivation

Conventional smart hospitals face hurdles such as limited connectivity, slow data transmission, and inadequate support for real-time applications. These issues hinder efficient remote care, timely decision-making, and seamless integration of advanced technologies like AI and IoT. 5G-based smart hospitals can address these challenges with ultra-fast data speeds, low latency, and massive device connectivity. 5G enables real-time telemedicine and remote surgeries by ensuring instantaneous communication between doctors and patients or robotic systems. It also supports the Internet of Medical Things (IoMT), allowing continuous monitoring and automated alerts for critical conditions. The high data capacity of 5G allows for rapid sharing of large medical files, such as MRI scans, facilitating faster diagnoses. Additionally, 5G improves hospital efficiency by enabling smart systems for managing resources, equipment, and staff, reducing operational delays. By enhancing speed, reliability, and device integration, 5G can resolve many challenges of conventional smart hospitals, significantly improving patient care. The integration of 6G technology in smart hospitals is motivated by the need to address the ever-growing demand for advanced, efficient, and personalized healthcare services. As healthcare systems face challenges such as an aging population, chronic diseases, and pandemics, the current infrastructure often falls short in delivering timely and effective care. 6G technology, with its unparalleled data transmission speeds, ultra-low latency, and massive connectivity, promises to revolutionize healthcare by enabling real-time monitoring, remote surgeries, and AI-driven diagnostics. The potential for enhanced communication between devices, patients, and healthcare providers can lead to more accurate and timely medical interventions. However, the adoption of 6G in healthcare also presents challenges, including concerns about data security, high costs of implementation, and the need for robust regulatory frameworks. Despite these hurdles, the opportunities offered by 6G technology—such as improved patient outcomes, reduced healthcare costs, and the facilitation of telemedicine—make it a critical component in the evolution of smart hospitals and the future of healthcare delivery. The structure of this paper is as follows: Section 1 provides the definition of smart healthcare with respect to 6G, the significance of smart healthcare in the modern era, the evolution and adoption of smart healthcare technologies with 6G, and the challenges facing the implementation of future 6G-centered healthcare facilities. In Section 2, we critically examine and analyze existing scholarly works on a specific topic. It provides a comprehensive overview of relevant research, identifying gaps, trends, and insights to inform and contextualize a new study or research endeavor. Includes an article published in this field. Section 3 focuses on the integration of several technologies into a 6G-based smart hospital. The benefits of 6G for smart hospitals are described, and the differences between 5G and 6G and their benefits owing to the differences in some quantitative performances are tabulated. Section 4 provides the perspective of advanced technologies, such as Internet of Things (IoT), explainable artificial intelligence (AI) in 6G, which will play an important role in future smart hospitals. The significance of prospective technology for 6G-based smart hospitals lies in its potential to revolutionize healthcare, as described in Section 4. With ultrafast communication, low latency, and massive device connectivity, 6G can enhance telemedicine, enable real-time diagnostics, support advanced robotics, and foster personalized patient care, ultimately improving healthcare efficiency and outcomes. Additionally, the architecture and different layers of advanced techniques are comprehensively discussed. Additionally, the challenges in 6G-based smart hospitals include ensuring robust cybersecurity to protect sensitive health data, addressing interoperability issues among diverse devices and systems, managing the massive influx of data, and overcoming potential ethical concerns related to advanced healthcare technologies. Finally, Section 5 outlines the integration of 6G technology in smart hospitals coupled with advanced techniques, which promises unprecedented improvements in healthcare. Furthermore, future work on security and privacy are highlighted. The contributions of the projected article are given below:

- The article explores how 6G enables seamless connectivity between IoT devices within smart hospitals, facilitating real-time data collection, remote monitoring, and automated management of medical equipment and patient health data.
- It highlights the role of 6G in enhancing AI capabilities, enabling faster processing of large datasets for diagnostics, personalized treatment plans, and predictive analytics, leading to improved patient outcomes.
- The article discusses the potential of 6G to strengthen blockchain applications in healthcare, ensuring secure and transparent management of patient records, reducing the risk of data breaches, and improving trust in data sharing across healthcare systems.
- It examines how 6G can revolutionize telemedicine by providing ultra-low latency and high-definition video streaming, enabling real-time, remote consultations, and even remote surgeries, thereby expanding access to quality healthcare services.
- The article delves into the use of 6G in supporting robotic systems for surgery, rehabilitation, and patient care within smart hospitals, offering precise, reliable, and safe medical procedures with minimal human intervention.
- It discusses how 6G enhances advanced data analytics by enabling the rapid processing of vast amounts of healthcare data, facilitating insights into patient health trends, resource allocation, and overall hospital management.

These contributions collectively underline the potential of 6G technology to transform healthcare delivery in smart hospitals, addressing challenges while opening new opportunities for enhanced, efficient, and secure.

## 2 Literature review

In the integration of 6G technology in smart hospitals, the starting point is to study the challenges and solutions deployment of advanced technologies such as IoT, AI, blockchain, telemedicine, robotics, and data analytics. These technologies serve as the input, enabling real-time patient monitoring, automated diagnostics, secure data management, and efficient remote consultations. The end point is the enhanced healthcare delivery system, characterized by improved patient outcomes, streamlined hospital operations, and robust data security. By leveraging 6G's ultra-fast connectivity and low latency, smart hospitals can achieve seamless integration of these technologies, leading to more personalized, efficient, and effective healthcare services. In this section, we present a critical and comprehensive analysis of existing literature (published academic works, articles, books, and other sources) on smart healthcare. It summarizes and synthesizes key findings, theories, and methodologies from existing studies and scholarly works. The rapid development of cellular connection systems has greatly accelerated the evolution and implementation of remote health monitoring and smart healthcare. The advanced long-term evolution (A-LTE) network now underpins modern healthcare systems. However, the development of smart hospitals and healthcare institutions is still nascent on a global scale. The introduction of the 5G network is set to elevate the standards of intelligent healthcare. Smart hospitals have distinct requirements compared to other applications in sectors like business, education, and general public services. This research evaluates how IoT and 5G will underpin the future “smart hospital,” anticipated to enhance throughput, efficacy, and coverage. The study focuses on implementing a hybrid detection technique for massive multiple-input multiple-output (MIMO) and non-orthogonal multiple access (NOMA) systems using QR decomposition and the M algorithm-maximum likelihood detection (QRM-MLD) combined with beamforming (BF). This approach aims to improve latency, spectrum efficiency, and network throughput in 5G systems. Additionally, the work provides a comparison between the proposed and traditional detection methods (10). The OFDM waveform method is pivotal in the context of smart hospitals, though it faces challenges such as bandwidth loss from guard bands, spectrum leakage, high Peak-to-Average Power Ratio (PAPR), and significant detection latency, which undermine its effectiveness. As 5G deployment becomes increasingly widespread globally, its advanced radio systems are expected to fulfill the comprehensive needs of smart healthcare facilities, which include high spectrum access, large capacity, great throughput, and low PAPR. The demand for bandwidth in digital hospitals has surged, necessitating networks that operate at peak efficiencies for tasks ranging from transmitting medical images to interfacing with wearable devices to ensure optimal patient care. The transition to digital hospitals with 5G connectivity will be critically shaped by the adoption of reliable transmission technologies. Current efforts are primarily focused on the implementation of innovative waveforms like NOM, UFMC, and FBMC systems. This work involves a detailed analysis and study of several parameters, including power spectrum density, bit error rate, capacity, and PAPR of both advanced waveforms and traditional OFDM techniques (11). This paper outlines the system architecture resulting from the integration of IoT technology in smart healthcare environments, detailing optimization considerations, challenges, and viable solutions. The technological infrastructure is divided into five distinct levels, with each layer's architecture, limitations, and methods thoroughly examined. This includes the size of the smart hospital, the scope of its intelligent computing capabilities, and the extent of its real-time big data analytics. The findings from the study are utilized to identify potential flaws in each tier of the smart hospital design model and suggest necessary adjustments. The document aims to serve as a comprehensive guide for managers, system engineers, and academics interested in optimizing the design of smart hospital systems, providing them with a clear road map for improvement (12). In this study, stochastic Petri nets were employed to evaluate the functionality and availability of a smart hospital system without the initial need for financial investment in actual equipment. These models are highly parametric, allowing for the adjustment of resource capacity, service times, failure and repair intervals, and the duration between failures. The initial model permits the configuration of several parameters, enabling the assessment of various scenarios. The investigation results highlighted the arrival rate as a crucial system characteristic. Particularly in scenarios with high arrival rates, a significant correlation was observed between Mean Response Time (MRT), resource utilization, and discard rate, demonstrating the impact of these factors on system performance (13). The article outlines the design principles for a health service platform app, including the health information perception terminal. With the advancement of big data, cloud computing, and information technology, the concept of smart healthcare has become increasingly significant. This new model, referred to as a health service platform, is gaining popularity and proving more practical compared to traditional healthcare services. The effectiveness of health monitoring is being enhanced through the use of wearable devices and various apps. There is a pressing need for an efficient and practical app-based health service platform that can cater to both older and younger populations, aiming to augment and streamline smart healthcare services (14). The article underscores the imperative for a robust and practical app-based health service platform that caters to both older and younger demographics, aimed at significantly enhancing and facilitating smart healthcare services. Building upon foundational concepts, it explores the design principles of the health service system and the health information perception terminal within this platform. The discussion extends to various aspects of the developed systems, including the unique contributions of each framework, detailed operational processes, performance outcomes, and the strengths and limitations inherent in these systems. Furthermore, the article addresses prevailing research challenges, critically evaluating the shortcomings of current systems and proposing prospective directions for advancement. This analysis is intended to furnish comprehensive insights into contemporary developments in smart healthcare systems, thereby equipping professionals with the knowledge necessary to make meaningful contributions to the field (15). This paper explores the advantages of cloud computing for healthcare applications, detailing IoT architectures, various communication protocols, sensor technologies, and both machine learning and deep learning techniques. It provides a comprehensive review of their respective benefits, limitations, and challenges. This study equips researchers with the necessary insights, enabling them to initiate their investigations by choosing a specific application or topic from the discussed methodologies. With strict adherence to security and privacy measures, cloud-based IoT and ML healthcare systems prove to be accurate and immensely beneficial for patients, caregivers, and hospital staff (16). The article explores potential challenges and market adoption barriers for IoT-based healthcare from both patient and professional perspectives. It addresses key issues such as interoperability, standardization, compensation, data storage, control and ownership, as well as trust and acceptability. To overcome these challenges, the paper suggests that contemporary healthcare will need to depend on policy support, regulation focused on cybersecurity, strategic caution, and the adoption of transparent policies within healthcare organizations to enable IoT solutions. Implementing IoT-based healthcare could significantly enhance population health and the efficiency of healthcare systems (17). As information technology advances, the concept of smart healthcare has increasingly captured interest. Smart healthcare revolutionizes the traditional medical system by leveraging cutting-edge information technologies such as the Internet of Things (IoT), big data, cloud computing, and artificial intelligence. These technologies enhance the efficiency, convenience, and personalization of healthcare services. In this review, the authors first outline the key technologies that underpin smart healthcare. We then explore the current state of smart healthcare across various significant domains. Lastly, the article addresses the current challenges faced by smart healthcare and offers recommendations for overcoming these obstacles (18). The article examines the potential of IoT technology to alleviate pressures on healthcare systems caused by an aging population and the rise of chronic diseases. It identifies standardization as a critical barrier to success in this area and proposes a standardized model for future IoT healthcare systems. The paper then reviews recent research on each element of this model, providing an evaluation of its benefits, drawbacks, and suitability for wearable IoT healthcare applications. Key challenges such as security, privacy, wearability, and low-power operation are addressed. The article concludes with recommendations for future research directions in this evolving field (19). The article addresses several barriers hindering the integration of IoT applications in healthcare. These include the generation of large volumes of non-essential data, concerns regarding patient data security and privacy, and the substantial costs associated with IoT adoption. It highlights the role of prosthetic sensors, which collect relevant data to aid real-time patient treatment, as a promising area for future research. This study underscores the potential of IoT to enhance healthcare delivery by focusing on specific, impactful applications (20). This research presents a fresh technique and develops an IoT-based prototype. Then, an elaborate theoretical framework was developed from this a cutting-edge prototype that demonstrates how the I-CARES system actually works. The system offers ongoing health status monitoring and analysis, as well as automatic, real-time emergency action that may ultimately save lives. It also gives information on pharmaceutical effects, side effects, and the patient's health state (21). This paper provides an in-depth examination of current research projects and the application of various technologies in smart healthcare systems. It delves into the latest studies, proposed methodologies, and existing solutions in the realm of smart healthcare, focusing on the implications of emerging technologies, applications, and challenges these systems face today and in the future. The aim is to present a comprehensive view of what IoT currently offers to the healthcare sector and what it promises for the future (22). This work meticulously examines the challenges at each stage of the big data handling process, which necessitate the use of advanced computing technologies for resolution. It argues that healthcare providers must be adequately equipped with the essential infrastructure to regularly generate and analyze big data, in order to develop strategies that enhance public health. Additionally, the paper highlights that contemporary healthcare institutions could revolutionize medical treatments and personalized medicine through a robust integration of biomedical and healthcare data (23). This paper addresses the privacy and security concerns associated with future healthcare applications, as highlighted in the study. The advent of fifth-generation networks is propelling the expansion of telehealth and smart healthcare solutions. Fundamental elements such as Quality of Life, Intelligent Wearable Devices, the Intelligent Internet of Medical Things, Hospital-to-Home transitions, and innovative business models are shaping the future of AI-driven intelligent healthcare. Many academic studies consider 6G technology a vital enabler of intelligent healthcare systems. Furthermore, Body Area Networks with integrated mobile health systems are evolving toward personalized health management and monitoring. Additionally, Extended Reality, a novel immersive technology, merges the real and virtual worlds, enabling enhanced interaction between computers, wearables, humans, and other machines (24). As the volume of daily-generated data expands in the 6G-enabled Internet of Medical Things (IoMT), the process of medical diagnosis becomes increasingly critical. This study, referenced in Wijethilaka et al. (25), develops a methodology aimed at enhancing prediction accuracy and facilitating real-time medical diagnosis within the 6G-enabled IoMT framework. The proposed approach integrates optimization techniques with deep learning methodologies to deliver precise and reliable outcomes. During the process, medical computed tomography images undergo preprocessing before being input into a sophisticated neural network designed to learn image representations and convert each image into a feature vector. Subsequently, a MobileNetV3 architecture is employed to further learn and refine the features extracted from these images (26). The 6G-Health project aims to foster precision technology development within the realm of sixth-generation mobile communications (6G) by integrating the expertise of communication engineering, medical engineering, and technical end users. The project's scope includes not only the development of specific 6G technological components but also the early identification and mitigation of market entry barriers, particularly focusing on operational elements, standards, and licensing issues. The technical framework encompasses emerging technologies that enhance network intelligence, innovative sensor connectivity for 6G, and efficient resource utilization and data processing strategies prior to their dissemination across various infrastructure levels. This paper will explore three medical applications of 6G: enhancing smart hospital operations, improving collaborative work environments, and enabling direct acquisition and transmission of bio signals from patients (27). The authors propose a Peak-to-Average Power Ratio (PAPR) reduction technique aimed at enhancing the efficiency of power amplifiers for 5G waveforms. This approach involves applying several algorithms to 5G waveforms, with their performance evaluated through PAPR curves. In the broader context, the study concludes that hospitals can leverage AI and IoT technologies to improve efficiency, reduce costs, and enhance patient care. By adopting these technologies, hospitals are positioned to improve patient outcomes and the overall health system's performance.

## 3 Smart hospital

A smart hospital, also referred to as a digital or intelligent hospital, is an example of how cutting-edge technologies, data-driven strategies, and patient-centered care have come together in the healthcare sector (10). It is a paradigm-shifting idea that seeks to integrate cutting-edge technologies and intelligent systems to optimize resource usage, improve operational efficiency, and improve patient outcomes. A sophisticated digital infrastructure that allows for seamless connectivity and data sharing across different hospital systems, equipment, and stakeholders is the foundation of a smart hospital (28). The two essential elements of smart hospitals are remote patient monitoring and telemedicine. Patients can obtain remote medical consultations, diagnoses, and follow-up care with the aid of communication technology. Healthcare professionals can remotely monitor patients' vital signs and medical issues using IoT connectivity and remote monitoring equipment (29). This reduces the need for hospital stays, enhances access to healthcare services, and permits continuous care, especially for patients with chronic illnesses (30). Smart hospitals prioritize patient empowerment and involvement using digital tools and technologies. Patients can access their health records, obtain personalized health advice, make appointments, and contact healthcare practitioners through mobile apps, patient portals, and wearable technology. These resources encourage patients to play an active role in their own care, help patients follow their treatment regimens, and help patients and healthcare teams work together. The idea of a “smart hospital” has a lot of potential, but it also has drawbacks.

The main obstacles are related to implementation costs, infrastructure needs, interoperability, and data protection. Furthermore, successful implementation depends on tackling the digital divide, negotiating regulatory frameworks, and guaranteeing that healthcare personnel integrate and accept new technologies (30). Establishing a connected healthcare environment is mostly dependent on Internet of Things (IoT) devices, cloud computing, and high-speed networks. Real-time data collection, monitoring, and analysis are made possible by these technologies, providing healthcare professionals with access to fast and reliable information for making decisions (31). Electronic health records (EHRs) are a fundamental component of smart hospitals. Electronic Health Records (EHRs) centralize and digitize patient data, including diagnoses, treatment plans, test results, and medical histories. Smart hospitals guarantee simple access to thorough and current information by digitizing patient data, which enhances care coordination and reduces medical errors. Artificial intelligence (AI) and data analytics are essential for a smart hospital operation. To extract valuable insights, advanced analytics algorithms can examine vast amounts of healthcare data, including patient records, medical imaging, and real-time monitoring data. AI-powered tools can help with tailored care, illness diagnosis, treatment planning, and clinical decision-making support. Healthcare professionals can make better judgments using machine learning algorithms that can recognize trends, forecast results, and offer recommendations. Robotics and automation are used in smart hospitals to improve patient care, increase productivity, and expedite procedures. Tasks, including pharmaceutical delivery, lab sample processing, and inventory management, are handled by robotic process automation (RPA). Surgeons are increasingly using robotic equipment to aid them in performing precise, minimally invasive surgeries known as robotic-assisted surgeries. Robotic caretakers can also assist with prescription reminders, patient monitoring, and mobility assistance (32). A smart hospital relies heavily on Internet of Things (IoT) devices to connect wearables, sensors, and medical devices. IoT-enabled gadgets gather health data, continuously check patients' vital signs, and send them to centralized platforms for analysis. Healthcare professionals can remotely monitor patient states, identify warning indications, and take immediate action through real-time monitoring. To ensure effective resource utilization, IoT devices also make asset tracking, inventory management, and medical equipment maintenance possible. Smart hospitals use cutting-edge technology, data analytics, and patient-centric strategies to bring about a paradigm shift in healthcare delivery. Smart hospitals are designed to improve patient care, increase operational efficiency, and change the healthcare experience of both patients and healthcare providers through seamless connectivity, intelligent technology, and real-time data analysis (33).

Differentiating itself from traditional hospitals, a smart hospital incorporates cutting-edge technology like IoT, AI, and big data to improve patient care, operational efficiency, and clinical outcomes. Networked equipment in smart hospitals facilitates real-time patient monitoring, enabling timely interventions. While automated technologies streamline administrative activities to reduce human error and wait times, AI-driven insights support tailored treatment plans and diagnostics. By extending care outside of the hospital, telemedicine and remote monitoring guarantee ongoing patient involvement. Conventional hospitals, on the other hand, are less able to provide the same degree of proactive, data-driven, and seamless healthcare services since they rely more on manual operations (34).

A number of enduring problems in healthcare, such as incorrect diagnosis, ineffective resource management, and patient safety, can be resolved by implementing 6G in smart hospitals. Personalized treatment regimens and improved diagnosis accuracy are achieved by advanced AI systems. Real-time information from networked devices optimizes the use of resources, easing congestion and improving patient flow. When it comes to prescribing medications and documenting clinical findings, automated technologies reduce human error. In addition, telemedicine and remote monitoring offer round-the-clock patient care, which lowers readmissions to hospitals and enhances the treatment of chronic illnesses, increasing overall health outcomes (35). The input refers to the existing or baseline infrastructure of conventional smart hospitals, including current technologies like 4G/5G networks, IoT devices, electronic health records (EHR), AI-driven healthcare solutions, and current limitations in terms of connectivity, data management, and real-time capabilities. It also includes the introduction of 6G technology and its core features such as ultra-low latency, high data transfer rates, AI integration, and seamless device connectivity. The output refers to the anticipated improvements and advancements brought by the integration of 6G technology in smart hospitals. This includes enhanced healthcare services like real-time remote surgeries, continuous patient monitoring with IoMT, AI-driven diagnostics, personalized treatments, and more efficient hospital operations. It also encompasses overcoming current challenges, such as data privacy, cybersecurity, interoperability, and cost-related hurdles.

### 3.1 Infrastructure requirements for 6G-based smart hospitals

Implementing 6G technology in the health sector, especially in developing countries, has considerable cost factors in terms of investment in large-scale infrastructure. Advanced hardware, including high-frequency antennas, fiber-optic cables, and edge computing devices, needs to be deployed for the rollout, which comes with a heavy installation and maintenance cost. Also, retrofitting existing infrastructure for ultra-low latency, high-speed communication, and extensive IoT integration comes with financial costs. Regulatory compliance, cybersecurity protocols, and staff training also contribute to the costs. In the developing world, scarce resources and poor infrastructure further compound these costs, requiring public-private partnerships and foreign aid. Phased rollout and reuse of existing 4G/5G infrastructure are cost-effective options that can help reduce upfront costs. Although having high initial costs, the long-term gains—enhanced health care access, increased telemedicine, and improved health outcomes—make the investment worthwhile, especially if underpinned by creative financing schemes and government subsidies. The successful implementation of 6G-based smart hospitals will require a comprehensive infrastructure that integrates advanced connectivity, IoT devices, AI technologies, and robust cybersecurity measures to deliver high-quality, personalized healthcare services efficiently and securely (36, 37).

- 6G connectivity: the backbone of any smart hospital would be its connectivity. 6G networks, expected to offer unprecedented speeds, low latency, and massive device connectivity, will be crucial. These networks will support high-definition video streaming for telemedicine, real-time monitoring of patients' vital signs, and seamless communication between IoT devices and AI systems.
- IoT devices: smart hospitals will heavily rely on IoT devices for various applications like remote patient monitoring, asset tracking, and environmental monitoring. These devices include wearable health trackers, smart beds, smart infusion pumps, and sensors for monitoring temperature, humidity, and air quality. With 6G, these devices can transmit data faster and more reliably, facilitating real-time decision-making by healthcare providers.
- AI and machine learning: advanced AI algorithms will analyze the massive amounts of data generated by IoT devices to provide insights for personalized patient care, disease prediction, and treatment optimization. These AI systems will require powerful computational infrastructure for processing data in real-time or near real-time, which could be facilitated by edge computing nodes within the hospital network.
- Robotic systems: robots will play a significant role in smart hospitals, performing tasks such as patient assistance, drug delivery, and disinfection. These robots will be equipped with sensors and cameras for navigation and interaction with patients and staff. High-speed, low-latency 6G connectivity will enable remote operation of robots by surgeons for telesurgery, particularly in emergency situations or in remote areas lacking specialized medical expertise.
- Optical fibers: to support the high bandwidth demands of 6G networks and ensure reliable connectivity throughout the hospital premises, optical fiber infrastructure will be essential. Fiber-optic cables offer greater bandwidth and immunity to electromagnetic interference compared to traditional copper cables, making them ideal for transmitting large volumes of data at ultra-fast speeds over long distances.
- Advanced cameras and imaging systems: high-resolution cameras and imaging systems will be deployed for various applications, including monitoring patient conditions, tracking medical equipment, and enhancing security. These systems will generate large amounts of data, which will need to be transmitted and processed efficiently using 6G networks and advanced AI algorithms.
- Cybersecurity measures: with the proliferation of connected devices and sensitive patient data being transmitted over 6G networks, robust cybersecurity measures will be critical to protect against data breaches, unauthorized access, and cyber-attacks. Hospitals will need to implement encryption protocols, access controls, and intrusion detection systems to safeguard patient privacy and ensure the integrity of medical data.
- Dense networks of small cells and energy consumption: The roll-out of 6G demands huge investments in infrastructure, especially in high-density small cell networks to enable the ultra-high speeds, low latency, and massive connectivity that 6G is expected to deliver. Small cells, scattered in urban and rural environments, will provide flawless coverage and connectivity by offloading traffic from conventional macro cells, hence alleviating congestion and enhancing network dependability. Their deployment, however, calls for vast physical infrastructure, such as the building of many base stations and antennas. Energy usage is yet another significant issue, with small cells and millimeter-wave and other high-frequency communication technologies requiring significant amounts of power to keep performance steady. The around-the-clock nature of these networks combined with sophisticated AI-based management means that effective use of power is needed to not overload the grid. To reduce these risks, the adoption of energy-saving technology such as low-power chips, solar-powered bases, and intelligent grid systems will be required. Moreover, improving network design by software-defined networking (SDN) and network slicing can further minimize energy usage with high performance.
- Cost implications: The infrastructure needed for 6G deployment is considerable, and it comes with high costs. Setting up a 6G network involves installing sophisticated hardware such as high-frequency antennas, small cells, massive MIMO systems, and fiber-optic backhaul links, all of which are costly to install and maintain. Moreover, creating a dense, distributed network of base stations to provide ubiquitous connectivity involves a huge investment in both urban and rural regions. The energy requirements for these systems, particularly edge computing and integration with AI, introduce additional cost complexity. In addition, maintaining cybersecurity and data privacy compliance comes at the cost of having strong security infrastructure, which adds to the overall expense. Although the advantages of 6G, including ultra-low latency, increased data rates, and enormous IoT support, are evident, the cost to governments, telecommunication companies, and healthcare systems could be high. Public-private collaborations and global funding will be necessary to balance these expenses and provide equal access to 6G technology.

Rolling out 6G infrastructure in rural and underdeveloped areas is challenging because of poor infrastructure, high expense, and a lack of technical skills. These regions usually do not have stable power grids, fiber-optic connections, and high-performance computing facilities, which hamper the implementation of 6G-based smart healthcare solutions. Moreover, the expense of installing small cells, massive MIMO antennas, and edge computing equipment is too high for governments and healthcare organizations. Socioeconomic conditions of low digital proficiency and constrained budgets for healthcare enlarge the digital gap further, restricting access to modern telemedicine, remote diagnosis, and AI-supported healthcare services. To tackle these constraints, affordable, scalable solutions will have to be given priority. Utilizing built-in 4G/5G infrastructure using network upgrades lowers the initial costs substantially. The use of low-power, solar-powered base stations can mitigate power limitations, and satellite-based internet services such as LEO constellations can provide coverage in remote locations. Furthermore, embracing open-access network architectures and software-defined networking (SDN) can reduce operating expenses and enable flexible infrastructure deployment. Public-private partnerships and international funding schemes must be promoted to finance infrastructure development and digital literacy programs. By adopting these measures, healthcare systems can close the connectivity gap so that 6G-enabled healthcare innovations reach rural and underdeveloped areas.

## 4 Sixth generation

The goal 6G wireless technology, which replaces 5G technology, is to improve mobile communication even more. While 5G concentrates on delivering greater speeds, reduced latency, and enhanced connectivity for Internet of Things devices, 6G is anticipated to completely transform these areas with even more breakthroughs. With terabits per second of data transport, 6G promises to outperform 5G by up to 100 times (38). By substantially reducing latency to microseconds, it will enable almost instantaneous communication. In addition, 6G will use cutting-edge technology like edge computing and artificial intelligence to enhance resource management and network performance. Furthermore, 6G will facilitate the creation of cutting-edge applications like sophisticated autonomous systems, immersive virtual reality, and augmented reality (39). Additionally, it will guarantee global digital inclusion by improving connections in underserved and distant locations. As a result, 6G will greatly increase the potential for wireless communication, outperforming 5G in terms of speed, latency, and technological integration. Global 6G standardization remains in its initial phase, with initiatives such as ITU, 3GPP, and national efforts of the U.S., China, South Korea, and the EU leading research and framework development. The emphasis lies in realizing ultra-low latency, high reliability, and massive connectivity to enable next-generation applications such as holographic communication, digital twins, and sophisticated healthcare systems. IoT, AI, and legacy healthcare systems will be integrated into 6G networks based on interoperable protocol, high-end edge computing, and slicing. Network management through AI will enhance resource utilization, forecast network faults, and make devices interoperable seamlessly. IoT healthcare devices, including remote monitoring and wearable devices, will interact in real-time to improve patient care. Legacy systems will require modular upgrades and backward-compatible interfaces to fit seamlessly. Cross-industry collaborations and joint standardization work will be pivotal to achieving safe, efficient, and ubiquitous uptake of 6G across healthcare and beyond (40).

Healthcare is changing because hospitals are implementing 5G technology, which makes data transfer and communication faster and more dependable. 5G networks currently provide much better speeds, lower latency, and increased connectivity than previous generations, all of which are essential for modern medical applications. Hospitals can monitor remote patients in real time and provide high-definition video consultations thanks to 5G telemedicine. This makes healthcare services more accessible, especially in underserved and rural areas. Moreover, 5G makes it easier to use IoT apps and cutting-edge medical devices. Smart beds, linked imaging systems, and wearable health monitors can all gather and send patient data continually, allowing for real-time monitoring and quick reactions to changes in a patient's condition. Massive amounts of data, including high-resolution medical images, can swiftly and effectively transfer to healthcare specialists for prompt diagnosis and treatment, thanks to the high bandwidth and low latency of 5G networks. The benefits of 5G extend to remotely operated medical equipment and robotic surgery. Surgeons can use robotic equipment to execute precise, minimally invasive operations even from remote locations because of 5G's ultra-reliable, low-latency transmission. This can help places without access to such resources by extending the reach of specialized medical knowledge (41). 5G has already made significant progress, but 6G has the potential to completely transform hospital operations. Even greater speeds—up to terabits per second—and microsecond-level latency will be possible with 6G technology, which is anticipated to be operational by the 2030s. This will facilitate real-time communication and very instantaneous data transfer, both of which are critical for vital medical applications. The combination of powerful edge computing and artificial intelligence (AI) will be one of the biggest developments with 6G (42). By processing enormous volumes of medical data locally at the network's edge, these technologies will lessen the need for data to go to centralized servers. This will assist AI-driven diagnosis, individualized treatment plans, and predictive analytics to identify health issues before they become serious by increasing the speed and efficiency of data analysis. Additionally, 6G will enable more complex and immersive telemedicine applications, such as augmented reality (AR) for remote surgeries, medical education, and holographic communication. These features will improve the caliber and reach of telemedicine, increasing its effectiveness and interactivity. Additionally, 6G's increased connection will aid in the development of a more extensive and cohesive healthcare ecosystem. It will ensure smooth data flow and integration across several healthcare systems by connecting an even wider range of medical equipment and sensors. This would allow for a holistic view of patients' health, which would improve care coordination and outcomes (43).

### 4.1 How to integrate 6G and smart health care

**Figure 1:**
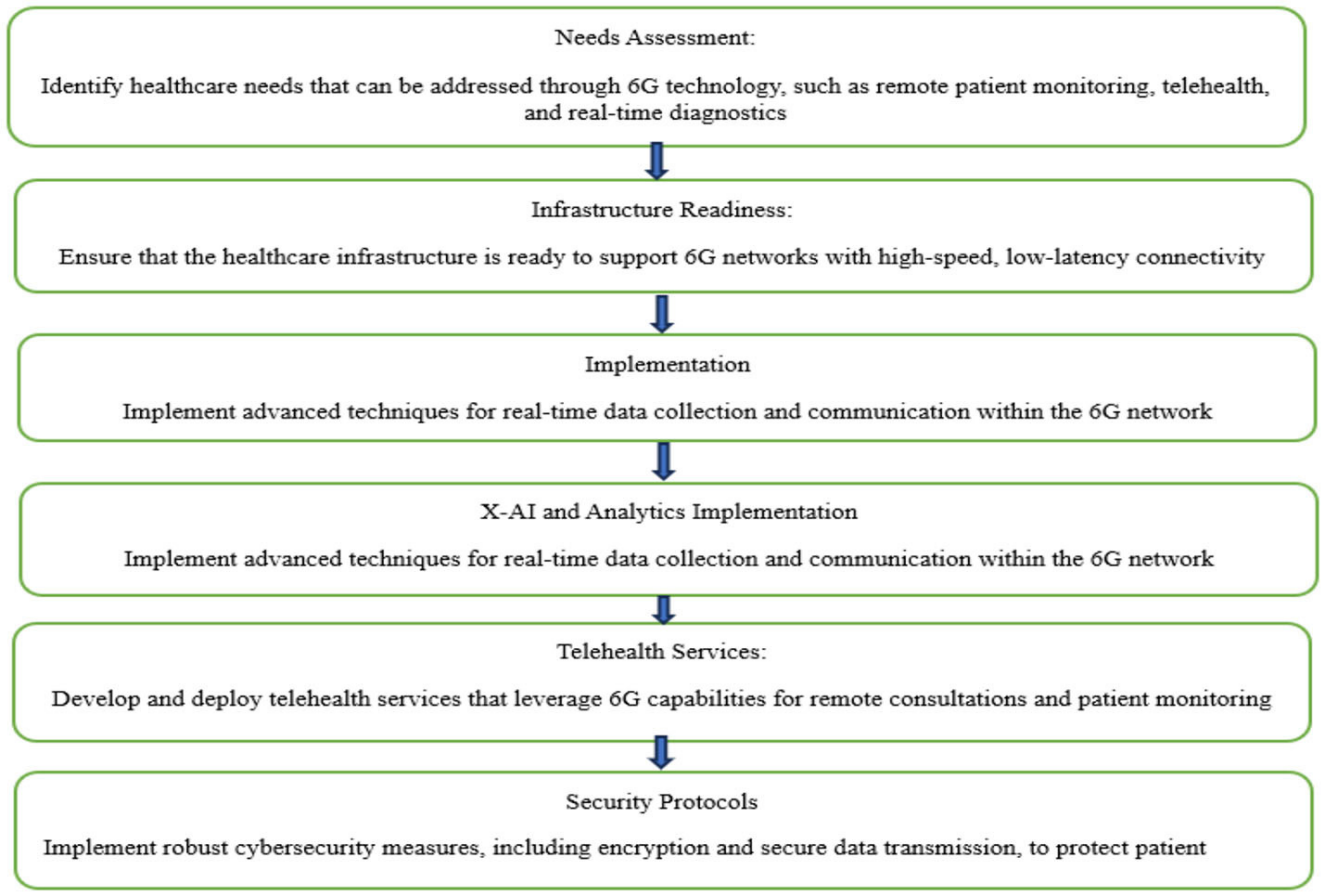
Flowchart of 6G integration with smart healthcare.

To transform patient care and improve healthcare systems, 6G technology must be strategically combined with healthcare breakthroughs and cutting-edge connectivity. 6G networks' blazing speed and low latency provide the groundwork for immediate connectivity, which makes it easier to integrate various healthcare sensors and equipment. With real-time data sharing made possible by 6G's fast connectivity, IoT devices can manage medication adherence, monitor patients' vital signs, and help healthcare providers make data-driven decisions more quickly. Furthermore, 6G's capacity to deliver high-quality low-latency video communication supports telemedicine applications. Telehealth services, including virtual consultations and remote patient monitoring, are becoming increasingly effective and widely available, particularly in underprivileged or isolated places (36). Protecting the privacy and security of sensitive healthcare data is critical. Strong cybersecurity safeguards protect patient data and ensure regulatory compliance within the 6G network. These protections include encryption and secure data transmission methods. Essentially, the combination of smart healthcare with 6G creates a dynamic environment in which cutting-edge medical solutions and dependable, quick connectivity can be achieved. This synergy opens the door to a revolutionary era in the provision of patient-centric care by improving the effectiveness, accessibility, and quality of healthcare services (44). A flowchart for integrating 6G and the smart hospital is shown in Figure 1.

**Figure 2:**
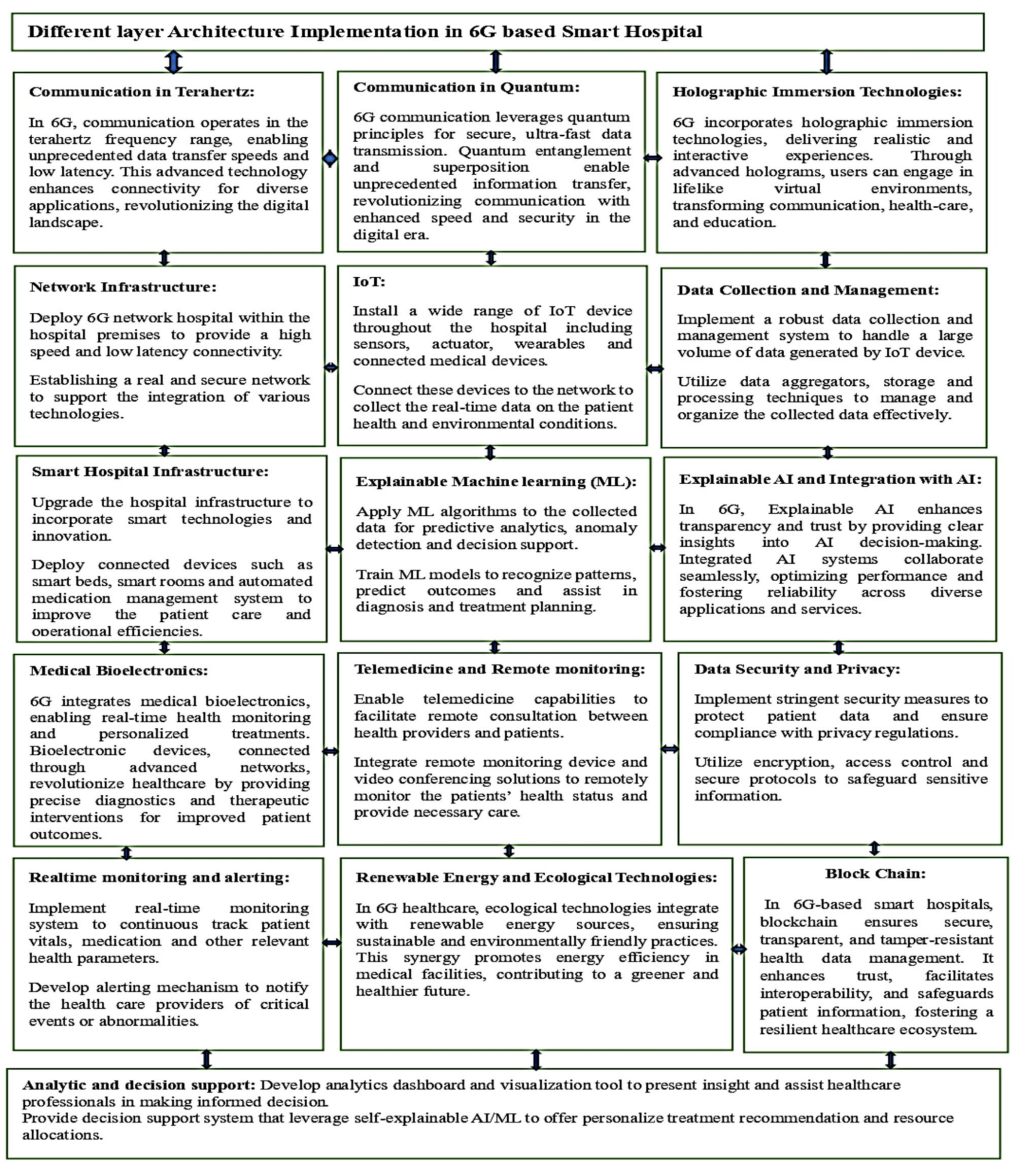
Different layers for 6G based smart hospital.

Investigating the use of various layer structures for cutting-edge approaches in real-world settings is crucial. Integrating 6G and smart healthcare involves leveraging the advanced capabilities of 6G networks to enhance healthcare services and enable innovative healthcare applications, as illustrated in Figure 2.

Integrating 6G technology with smart healthcare involves a systematic approach to leveraging the capabilities of advanced connectivity and healthcare innovations (2, 45–49).

- **Remote patient monitoring**: 6G technology, known for its low latency and high-speed connectivity, facilitates real-time remote patient monitoring. Healthcare providers can employ connected devices to continuously monitor various patient metrics, such as vital signs, medication adherence, and overall health status from a distance. The collected data are instantly transmitted to healthcare professionals, enabling them to make well-informed decisions and deliver prompt interventions. The integration of remote patient monitoring systems with 6G networks guarantees an uninterrupted and reliable data flow, thus supporting proactive healthcare management.
- **Telemedicine and virtual consultations**: 6G enables high-quality video conferencing and real-time communication, making telemedicine and virtual consultations more accessible and efficient. Healthcare providers can offer remote consultations, diagnosis, and treatment recommendations to patients located anywhere, eliminating geographical barriers and improving access to healthcare services. Integrating telemedicine platforms with 6G networks ensures seamless and reliable communication, high-quality video streaming, and secure data transmission. 6G's ultralow latency and high connectivity will greatly enhance telemedicine and robotics by supporting near-instant data transfer and real-time reaction, vital to applications in critical healthcare. In telemedicine, physicians will be able to remotely consult with patients within negligible delay, increasing diagnostic accuracy and patient treatment even for poorly served or rural regions. Real-time video streams, high-definition imaging, and advanced diagnostic information will be easily transferred, permitting more effective remote monitoring and diagnosis.
- **Renewable energy and ecological technologies**: these play a pivotal role in 6G-based smart hospitals, contributing to sustainability and environmental consciousness. The integration of renewable energy sources, such as solar panels and wind turbines, ensures a reliable and eco-friendly power supply and reduces the carbon footprint of these advanced healthcare facilities. Energy-efficient designs and smart grid technologies optimize energy consumption, aligning with green initiatives. Ecological technologies, including green building materials and sustainable infrastructure, further enhance the environmental responsibility. By prioritizing renewable energy and ecological practices, 6G smart hospitals not only reduce operational costs but also demonstrate a commitment to a healthier planet, aligning technological advancements with ecological sustainability in the pursuit of cutting-edge healthcare solutions.
- **Blockchain:** this ensures confidentiality, openness, and integrity of medical data, which is essential in 6G-based smart hospitals. Blockchain technology improves patient privacy and protects medical records by utilizing tamper-resistant and decentralized ledgers. Blockchain-based smart contracts protect and automate several healthcare operations, including supply chain management and billing. Furthermore, blockchain promotes interoperability, making it possible to exchange data securely and effortlessly for various health care systems and devices. Smart hospitals build a solid foundation for data accuracy, trust, and efficient operation by integrating blockchain into 6G networks. This eventually increases the overall effectiveness and dependability of healthcare services.
- **Robotics:** 6G's ultralow latency and increased connectivity will revolutionize robotics to a great extent by facilitating real-time communication and exact control over robot systems, particularly in sophisticated applications such as surgery, manufacturing, and remote control. 6G's ultra-low latency of usually sub-millisecond order ensures that instructions sent to robots are carried out with little delay, essential for processes demanding high accuracy and coordination. In robot surgeries, for example, this means surgeons can manipulate robotic arms in near-instantaneous feedback, minimizing the chance for mistakes and enhancing patient outcomes. And the improved connectivity of 6G and the capacity to carry massive IoT networks will also make the smooth integration of different devices, sensors, and robots possible, allowing for collaborative tasks and autonomous decision-making. With 6G's enormous throughput, robots are able to send data-rich information, such as high-definition video or 3D mapping, uninterrupted, further propelling autonomous robotics in telepresence, industrial automation, and healthcare.

### 4.2 Challenges in 6G based smart hospital

As with any new technology, the development and deployment of 6G faces challenges and considerations. The implementation of 6G will require substantial investment in infrastructure, including new antennas, base stations, and network equipment. Table 1 indicate the challenges faced by 5G and 6G based smart hospital (50, 51).

**Table 1:** Challenges of 6G and 5G in smart hospitals.

**Parameters**	**5G**	**6G**
Network dependability and coverage:	•The challenge is in providing dependable and stable 5G service over the hospital's grounds, particularly in difficult-to-reach locations like basements and specialized medical units. •Real-time monitoring and vital healthcare applications may be interfered with by uneven coverage.	•The challenge lies in creating and deploying communication devices that use terahertz frequencies to transfer data at a quicker rate. •Ensuring dependable communication at these higher frequencies and overcoming obstacles related to signal attenuation.
Latency	•While 5G brings low latency, it is important to sustain low latency continuously for applications such as real-time patient monitoring or remote surgery. •High latency can affect the real-time responsiveness of vital medical applications and jeopardize the efficacy of remote healthcare services.	•The challenge lies in achieving and sustaining ultra-low latency to facilitate new applications like augmented reality (AR) for surgical help and medical training. •The real-time responsiveness necessary for vital medical treatments may be hampered by high latency.
Security issues:	•Handling cybersecurity issues brought on by the rise in connected devices and the network's transmission of private patient data. •Unauthorized access to patient records resulting from security breaches puts patient privacy and the accuracy of medical data at danger.	•Keeping an increasingly data-intensive and networked healthcare environment secure and private is a challenge. •Cybersecurity risks have the potential to jeopardize private patient information and interfere with medical operations.
IoT device integration:	•The challenge is in efficiently incorporating a wide variety of IoT gadgets and medical apparatuses into the 5G network. •The potential advantages of connected devices in healthcare may be limited by poor integration, which might impede data flow and interoperability.	•Ensuring smooth interoperability across various devices becomes a crucial concern as the quantity and variety of IoT devices in smart hospitals rise. Effective integration may be hampered by the absence of common data formats and communication protocols among different device kinds and manufacturers. •Healthcare providers would find it challenging to integrate new IoT devices into the 6G network in the absence of defined protocols, which could result in inefficiencies, data silos, and possible disruptions in the flow of operational and patient data.
Scalability	•The challenge is in making sure the 5G network can expand to handle the growing volume of data and the growing number of linked devices in smart hospitals. •Impact: Poor performance and network congestion might result from inadequate scalability.	•The expansion of wearables, medical sensors, and IoT devices in smart hospitals presents a major scalability barrier for 6G networks due to the sheer volume of linked devices. Every device needs a dependable connection, and network scalability becomes more important as the number of devices rises. •The performance of vital healthcare applications and services can be negatively impacted by inadequate scalability, which can cause network congestion, lower data transfer rates, and possible communication disruptions. •Smart hospitals generate enormous amounts of data due to the growing demand for real-time video streaming, high-resolution medical imaging, and other data-intensive applications. 6G networks face scaling issues in effectively managing this spike in data traffic and guaranteeing the efficient transmission of big datasets.
Regulatory and ethical considerations:	•5G networks are used by smart hospitals to handle and transfer enormous volumes of patient data, including private medical records. It is crucial to comply with legal requirements and provide the greatest levels of data security and privacy. Concerns around illegal access, data breaches, and the possible exploitation of patient information are raised by the interconnectedness of the systems and equipment in smart hospitals. •Ignoring these privacy and security issues may have unethical and legal repercussions, damage patient confidence, and result in noncompliance with regulations.	•The challenge is addressing moral questions about the application of cutting-edge medical technology, such AI-driven diagnosis and therapy. •Establishing trust in the use of 6G technology in healthcare settings requires adherence to legal obligations as well as ethical norms.
Tailored healthcare services:	•Creating and deploying 5G networks that are capable of meeting the many and unique requirements of the healthcare industry. Within a smart hospital, various medical specialties and departments can need different network setups and capabilities to serve their own devices and apps. •Ignoring this issue could lead to subpar performance for some healthcare services, which would reduce the potential advantages of customized and specialized solutions. It could result in ineffective care delivery of specialist treatment.	•The challenge lies in creating 6G networks that are specifically designed to meet the demands of healthcare applications. •Impact: The efficacy of cutting-edge healthcare services and solutions may be restricted by inadequate personalization.

Spectrum allocation and regulatory frameworks need to be established to facilitate the efficient and secure deployment of 6G networks. To understand why 6G is required, it is important to consider the limitations and evolving requirements of existing wireless communication technologies, such as 5G. Although 5G has brought significant improvements over its predecessors, it still faces certain challenges that 6G aims to address. The following are some key reasons why 6G is required (52, 53):

- **Expanding data traffic**: as the proliferation of connected devices, IoT applications, and data-intensive services continues to drive an exponential increase in data demand, 6G technology has been poised to meet this challenge. It is anticipated to deliver significantly higher data rates and capacities, which are essential for managing the growing volume of data traffic. This advancement will facilitate seamless streaming of ultra-high-definition content, enhance immersive experiences in virtual and augmented reality, and support emerging technologies that depend on massive data transfers.
- **Ultra-low latency**: certain applications and services require real-time responsiveness with minimal delays. Industries such as autonomous vehicles, remote surgery, and industrial automation rely on ultralow-latency networks to enable time-critical operations. 6G aims to further reduce latency, enabling instantaneous communication and unlocking new possibilities for mission-critical applications.
- **Massive device connectivity**: the rise of IoT devices and the vision of a fully connected world necessitate networks that can handle an enormous number of simultaneous connections. 6G supports a large number of devices per unit area, enabling seamless connectivity for smart homes, smart cities, and various IoT applications. This will enable the efficient management of billions of connected devices and unlock the potential of a hyperconnected society.
- **Transformative applications**: 6G integrates various cutting-edge technologies such as AI, machine learning, and quantum computing. These technologies require networks with enhanced capabilities to effectively process and transmit data. With 6G, transformative applications such as AI-driven smart assistants, advanced healthcare solutions, and intelligent transportation systems can become a reality, fostering innovation and improving quality of life.
- **Future-proofing technology**: developing 6G networks is a proactive approach to future-proof communication infrastructures. This allows us to stay ahead of the emerging technologies and unforeseen demands. By investing in 6G research and development, we can ensure that our networks are ready to meet the challenges and requirements of the next decade and beyond.

The need for 6G arises from the ever-growing demand for faster speeds, higher capacity, ultralow latency, massive device connectivity, and the integration of transformative technologies. 6G will empower industries, enable new applications, and provide a foundation for a more connected and technologically advanced future.

### 4.3 How 6G will benefit the health industry

The advent of 6G, the latest in the evolution of wireless communication networks, is set to revolutionize the healthcare industry by transforming the delivery of healthcare services. Integrating 6G technology into smart hospitals promises transformative advancements in healthcare, enabling faster, more reliable, and intelligent medical services. One of the key opportunities lies in ultra-low latency and high data rates, supporting real-time applications like remote surgeries and advanced telemedicine. Enhanced connectivity between medical devices and systems will enable seamless data sharing, improved diagnostics, and personalized treatments through AI-driven analytics. Additionally, 6G's support for massive machine-type communications (mMTC) will boost the deployment of Internet of Medical Things (IoMT) devices, allowing continuous patient monitoring, early disease detection, and automated interventions. However, several challenges need to be addressed. Ensuring robust cybersecurity measures is critical due to the sensitive nature of medical data. Managing data privacy in compliance with strict healthcare regulations, while maintaining system integrity is complex. Furthermore, the cost of upgrading hospital infrastructure to accommodate 6G networks may be prohibitive for many institutions, particularly in developing regions. Another concern is interoperability with existing medical devices and systems, requiring seamless integration for effective functionality. Additionally, managing the energy consumption of 6G networks and devices, as well as ensuring the ethical use of AI and big data in decision-making, poses significant hurdles. Overall, while 6G has immense potential to revolutionize healthcare delivery, addressing these technical, financial, and ethical challenges is essential to fully harness its benefits in smart hospitals. Table 2 indicates the advantages of 6G over 5G. Table 3 shows the benefits of 6G over 5G based smart hospitals (54).

**Table 2:** 6G and 5G technologies for smart hospitals.

**Key technologies in 5G**	**Key technologies in 6G**
5G New Radio (NR): The standard for 5G networks' air interface is called 5G NR. For a variety of devices, it provides reduced latency, increased connectivity, and quicker data rates.	Communication in terahertz: Description: Extremely fast data rates and accurate sensing are made possible by terahertz frequencies, which may be employed in 6G. Terahertz communication has the potential to improve imaging technology in smart hospitals and enable more precise diagnosis.
Slicing a network: Network slicing within the expansive 5G infrastructure enables the creation of virtual, isolated networks tailored to specific needs. In smart hospitals, network slicing allows for the segmentation of the network to cater distinctively to various healthcare services and applications. This technology provides the flexibility to allocate resources efficiently, ensuring that each healthcare function receives the necessary network support to operate optimally.	Explainable AI and integration with AI: It is anticipated that 6G would further incorporate AI into the communication network. In healthcare applications, explainable AI—which offers openness in AI decision-making—may be essential. AI systems may become more prevalent in healthcare administration, treatment planning, and diagnosis.
Enormous IoT connectivity: In smart hospitals, the implementation of medical sensors, wearables, and other connected devices is made easier by 5G's huge support for IoT devices. Real-time 4data collecting and monitoring are made possible by this technology.	Communication in quantum: One prospective 6G feature is quantum communication, which provides higher security while sending private medical information. It might be used to safeguard patient privacy and maintain the accuracy of medical records by securing communication routes inside smart hospitals.
Cutting edge computing: Edge computing lowers latency by bringing processing power closer to the data source. Edge computing improves the functionality of healthcare apps in smart hospitals, including real-time diagnostics and remote patient monitoring.	Medical bioelectronics: The field of bioelectronic medicine focuses on manipulating the electrical impulses produced by the body using electronic equipment. 6G could make it possible for smart hospitals to use closed-loop systems and cutting-edge bioelectronic therapies for individualized and accurate treatment plans.
Virtual reality (VR) and augmented reality (AR): High-bandwidth and low-latency connections are made possible by 5G, which makes immersive technologies like AR and VR possible. These tools can be applied to surgery planning, patient education, and medical training in smart hospitals.	Holographic immersion technologies: It is possible that 6G will enable cutting-edge holographic technologies, enabling realistic and engrossing 3D experiences. This has the potential to improve patient education, team-based surgery, and medical training.
	Renewable energy and ecological technologies: The focus of 6G is anticipated to be on sustainable technology and energy efficiency. By using energy harvesting technology to power IoT devices, smart hospitals can lessen the environmental effect of their healthcare operations.
	Blockchain: Blockchain guarantees safe, unhackable data interchange and storage in 6G-based smart hospitals. It increases data integrity, uses smart contracts to automate procedures, and fosters interoperability to increase efficiency and trust in healthcare operations.

**Table 3:** Additional benefits of 6G smart hospital over 5G based smart hospital.

**Parameters**	**6G Benefits**
Extremely high data speeds	Compared to 5G, 6G is anticipated to offer even faster data speeds. Faster transmission of big medical datasets, high-resolution imaging, and real-time video feeds may be made possible by this exceptionally high data rate capacity. Applications for collaborative healthcare, remote diagnostics, and telemedicine can all be greatly improved by this.
Precision medical applications of terahertz communication	New opportunities in precision medicine may arise from 6G's prospective feature, terahertz communication. Advanced diagnosis and treatment planning are made possible by the highly accurate sensing and imaging made possible by terahertz frequencies. This may result in more focused medical treatments and individualized treatment plans.
Improved communication in real time	Applications like augmented reality (AR) and virtual reality (VR) can function more smoothly and responsively because to 6G networks' extremely low latency. This could facilitate collaborative virtual consultations, immersive medical training, and AR-assisted procedures in the healthcare setting.
Extensive device networking for internet of things healthcare	6G can effectively enable the widespread adoption of IoT devices in healthcare thanks to its even higher connection density. This covers a broad range of wearables, monitoring tools, and medical sensors. As a result, patient monitoring, preventive care, and overall healthcare management are all improved by a more extensive and integrated healthcare ecosystem.
Advanced integration of AI	Advanced artificial intelligence (AI) technology integration can be made easier by 6G networks. This covers machine learning apps, predictive analytics, and AI-driven diagnostics. More sophisticated healthcare solutions may result from the smooth interaction between devices and AI algorithms made possible by the improved connectivity and data rates.
Green and sustainable communication	Energy efficiency and environmentally friendly communication technologies are anticipated to be prioritized in 6G as environmental sustainability becomes a bigger priority. 6G-enabled smart hospitals might use less energy, which would lessen the negative effects of healthcare operations on the environment.

Additionally, with its faster speed, lower latency, higher capacity, and integration of transformative technologies, 6G is poised to significantly benefit the smart healthcare industry in numerous ways. The potential benefits of 6G in smart healthcare are as follows (41, 55):

- **Enhanced connectivity and remote care**: 6G technology will significantly enhance connectivity, enabling seamless communication between healthcare providers and patients irrespective of geographical barriers. With its high-speed and reliable connections, 6G will significantly expand the scope of remote care services. This will allow physicians to monitor patients remotely, conduct telemedicine consultations, and offer real-time guidance during emergencies. Patients in remote areas gain access to specialized healthcare without the need for physical travel, thus ensuring equitable access to high-quality medical services. Enhanced connectivity in 6G will revolutionize remote care in smart healthcare by providing ultra-reliable, high-speed communication, enabling seamless, real-time patient monitoring, and consultation. With the support of massive IoT devices, 6G will facilitate the integration of a wide array of health monitoring tools, such as wearable sensors and remote diagnostic equipment, into the healthcare ecosystem. This will allow healthcare providers to monitor patients continuously, even from remote locations, improving outcomes for chronic conditions and reducing the need for in-person visits. AI algorithms will leverage this real-time data to offer personalized care recommendations, and telemedicine consultations will be nearly as efficient as in-person visits, thanks to 6G's low latency. Furthermore, 6G will expand access to healthcare for underserved populations, including those in rural areas, by enabling high-quality remote healthcare services that were previously unfeasible due to connectivity limitations. Enhanced connectivity ensures that patients receive timely care, regardless of their location.
- **Internet of medical things (IoMT) advancements**: IoMT refers to the interconnected network of medical devices and sensors. 6G's higher capacity and massive device connectivity will greatly advance the IoMT ecosystem, enabling a multitude of devices to seamlessly communicate and exchange data. This will result in more accurate patient monitoring, efficient data collection, and improved decision making for healthcare providers. With 6G, wearable devices, implantable sensors, and smart medical equipment operate seamlessly, providing real-time health data for better diagnosis, personalized treatment plans, and proactive healthcare management.
- **Real-time monitoring and emergency response**: 6G's ultra-low latency and high-speed connectivity will enable real-time monitoring of patient health conditions and instant communication in emergency situations. Wearable devices equipped with biosensors and vital sign monitors continuously collect data that can be instantly transmitted to healthcare professionals. This will enable timely intervention and rapid response in critical situations, potentially saving lives. Furthermore, emergency responders have access to live video streams and real-time data from accident sites, enabling them to make informed decisions and provide immediate medical assistance. 6G technology can significantly benefit different healthcare environments outside the typical hospital setting, such as rural healthcare, home care, and emergency response networks. In rural settings, where specialized care access is typically lacking, 6G's ultralow latency and high data rates will provide real-time telemedicine consultations and remote monitoring capabilities, enhancing health care accessibility and minimizing the need for lengthy transportation. With increased connectivity, healthcare professionals are able to remotely monitor their patients through wearable devices, diagnose ailments in real-time, and offer tailored care, closing the rural-urban healthcare divide. For home care, 6G may facilitate round-the-clock patient monitoring, making it possible to integrate smart home appliances and IoT-based health monitors that feed in constant streams of data into the hands of healthcare professionals. This promotes proactive management of health and early intervention, minimizing readmission to hospitals and enhancing patient outcomes. Additionally, decision support systems based on AI may aid caregivers through real-time feedback on a patient's status. In emergency response systems, 6G's huge connectivity and ultra-high reliability will allow first responders, hospitals, and command centers to coordinate more speedily and efficiently. Real-time data exchange, including live video streams and patient medical records, will support situational awareness, accelerating critical decisions in emergencies such as accidents or natural disasters. In general, 6G will enable more decentralized, efficient, and personalized medicine, enhancing outcomes and minimizing disparities across diverse healthcare environments.
- **Artificial intelligence (AI) integration**: the Integration: The 6G with AI technologies will drive significant advancements in smart healthcare. AI algorithms are capable of analyzing vast amounts of medical data collected through connected devices and electronic health records, aiding healthcare providers in making accurate diagnoses, performing predictive analytics, and offering personalized treatment recommendations. AI-powered virtual assistants and chatbots can provide support to 24/7 patients, respond to inquiries, and deliver basic medical advice. Moreover, AI-based systems for image recognition and interpretation will significantly enhance the analysis of medical imaging, thereby improving both the speed and accuracy of diagnosis.
- **Augmented reality (AR) and virtual reality (VR) applications**: with Owing to their high bandwidth and low latency, 6G will facilitate immersive AR and VR experiences in healthcare. Surgeons benefit from AR overlays during complex procedures that provide real-time guidance and detailed visualization of critical anatomical areas. In addition, medical education and training will see significant enhancements through VR simulations, enabling students to practice procedures in highly realistic virtual settings. AR and VR also play a crucial role in patient education, offering individuals a more interactive and engaging way to understand their medical conditions and treatment options.
- **Precision medicine and personalized healthcare**: the integration of AI, big data analytics, and advanced connectivity offered by 6G will enable the adoption of precision medicine approaches (2). By analyzing extensive datasets encompassing genomic information, patient histories, lifestyle factors, and real-time health data, healthcare providers can offer personalized treatment plans that are uniquely tailored to each individual's needs. This data-driven method enhances healthcare outcomes, minimizes adverse drug reactions, and boosts overall patient wellbeing.
- **Efficient healthcare resource management**: 6G's advanced capabilities will support the efficient management of healthcare resources. Through real-time monitoring and predictive analytics, healthcare providers can anticipate demand, optimize bed allocation, and allocate medical personnel more effectively. The seamless exchange of data between hospitals, clinics, and pharmacies will streamline inventory management, reduce waste, and ensure the availability of essential medications and supplies.
- **Enhanced patient engagement and self-care**: 6G will empower patients to actively manage their health through innovative healthcare applications and services. Mobile apps and wearable devices connected to 6G networks offer real-time health monitoring, personalized health recommendations, and reminders for medication adherence. Patients can conveniently access their health records, schedule appointments, and communicate with healthcare providers through secure mobile platforms.
- **Data security and privacy**: With the integration of advanced security measures and encryption protocols, 6G prioritizes data security and patient privacy. Robust authentication mechanisms and secure data transmission protocols ensure the confidentiality and integrity of sensitive health information and build trust among patients and healthcare providers.

6G offers several advantages over 5G, including a faster speed, lower latency, enhanced capacity, transformative technologies, and expanded coverage. However, it also presents challenges such as longer implementation timelines, higher infrastructure costs, spectrum considerations, compatibility issues, and regulatory/security considerations. These factors need to be carefully addressed as the development and deployment of 6G progresses in the coming years. 6G has the potential to revolutionize the smart healthcare industry by providing enhanced connectivity, enabling remote care services, advancing the IoMT ecosystem, enabling real-time monitoring and emergency response, integrating AI and VR/AR technologies, facilitating precision medicine, optimizing resource management, empowering patient engagement, and prioritizing data security and privacy. These advancements will contribute to improved healthcare outcomes, increased access to quality healthcare services, and a more efficient and patient-centric healthcare system (56). Cybersecurity and data privacy threats in 6G-enabled healthcare systems are paramount issues, considering the confidentiality of medical information and the growing attack surface created by IoT devices and remote care technologies. To counter these threats, embracing a zero-trust architecture (ZTA) is imperative, verifying users, devices, and applications continuously irrespective of location. ZTA enforces least-privilege access and employs multi-factor authentication (MFA) and real-time anomaly detection to block unauthorized access. Homomorphic encryption (HE) also provides a strong solution by allowing computations on encrypted data without decryption, maintaining privacy throughout data processing. Using blockchain for tamper-proof health records and deploying AI-powered threat detection systems can also increase security. Ongoing security audits, employee training, and adherence to global standards such as HIPAA and GDPR are of paramount importance. Cooperative working between healthcare providers, technology creators, and regulators will be instrumental in the creation of responsive, robust defenses that safeguard patient information while not diminishing the efficacy of sophisticated 6G uses (57). 6G's increased connectivity and device integration are poised to revolutionize sectors like healthcare by enabling faster, more efficient communication and data exchange. However, this hyperconnectivity also introduces significant vulnerabilities, particularly concerning cybersecurity. As healthcare infrastructures become more reliant on interconnected devices—such as IoT-enabled medical equipment, wearables, and cloud-based systems—the attack surface for cybercriminals expands exponentially. In a 6G environment, where billions of devices communicate seamlessly, malicious actors could exploit weaknesses in both hardware and software to gain unauthorized access to sensitive health data or disrupt critical operations.

For example, cyberattacks targeting hospital systems could compromise patient care by manipulating real-time data from life-saving equipment, leading to inaccurate diagnoses or treatment errors. The incorporation of artificial intelligence (AI) in healthcare also raises the level of complexity, and it may be simpler for the attackers to tamper with algorithms, leading to defective decision-making. The wide adoption of cloud computing and edge devices in 6G networks also raises the risk of data breaches or ransomware attacks because healthcare organizations will find it challenging to secure massive amounts of data across different platforms. The sheer volume and complexity of interconnected devices in 6G networks could make traditional security protocols less effective, requiring the development of advanced cybersecurity solutions. Without robust defense mechanisms in place, the healthcare sector faces heightened risks, jeopardizing not only patient privacy but also the very integrity of the healthcare system.

## 5 Key technologies in 6G based smart hospital

5G improves smart hospitals by offering fast, low-latency connectivity, which makes effective data transfer and real-time monitoring possible. Building on this base, 6G will revolutionize patient care, treatment, and diagnosis by providing quantum communication, holographic interfaces, and powerful AI. When combined, these technologies enable smart hospitals to become extremely intelligent, responsive, and flexible healthcare ecosystems. Table 3 shows the differences between 5G and 6G key technologies in smart hospitals (58).

### 5.1 Internet of Things (IoT) in 6G based smart hospital

**Figure 3:**
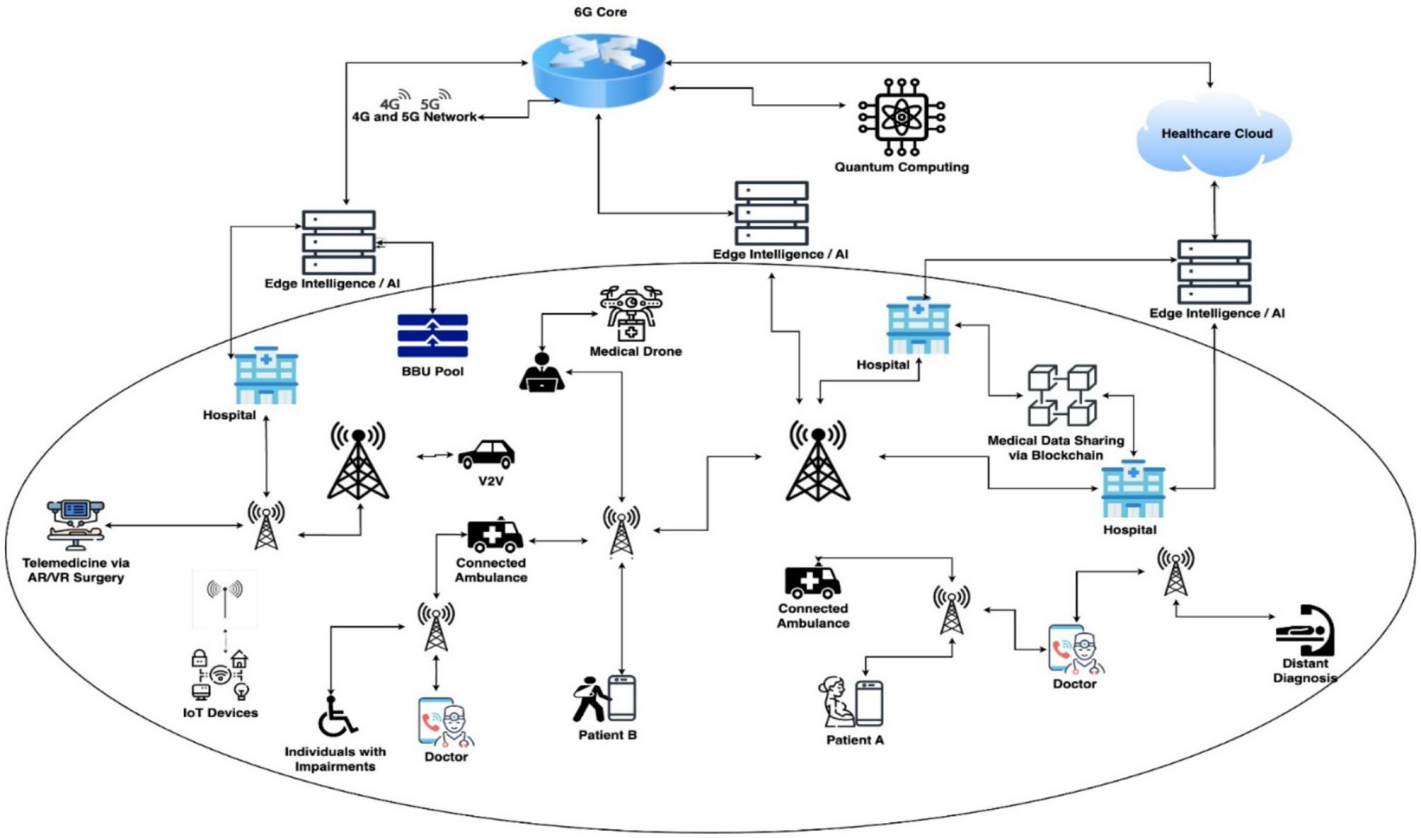
IoT empowered 6G smart hospital.

The architecture of a 6G-enabled IoT smart hospital, which aims to integrate real-time data processing, intelligent decision-making, and enhanced networking as shown in Figure 3. The architecture's central component is a dense network of IoT devices that run on 6G's incredibly rapid and low-latency network. Examples of these devices include connected imaging equipment, smart beds, and wearable health monitors (59). These devices ensure rapid processing and analysis near the data source by continuously gathering and transmitting patient data to edge computing nodes within the hospital. This configuration enables real-time monitoring, prompt notifications, and pre-emptive responses (60). Sophisticated AI algorithms analyze the data for predictive analytics, customized treatment plans, and diagnostics. Centralized cloud platforms store and manage large volumes of healthcare data, facilitating seamless integration and accessibility for healthcare providers. Massive MIMO and sophisticated beamforming are included in the network architecture to improve capacity and connectivity. Improved security protocols safeguard patient information while guaranteeing adherence to strict healthcare laws. This intelligent, integrated infrastructure transforms the hospital's operations and enhances operational effectiveness and patient care (59).

#### 5.1.1 IoT sensors in smart hospital

Hospitals use a variety of IoT sensors to manage resources, monitor patients, and increase productivity. With ultra-fast speeds, low latency, and strong security, 6G connectivity dramatically improves the functionality and dependability of these sensors in a smart hospital setting, enhancing patient care and safety (11). The list below includes common hospital sensors and discusses how 6G connectivity enhances their usability (61).

- Wearable health monitors sensors: take temperature, blood pressure, oxygen saturation, heart rate, and other vital signs. 6G's high-speed, low-latency connectivity ensures real-time data transfer, enabling quick analysis, and reaction. 6G enhances security measures to prevent breaches of crucial patient data.
- Smart beds sensors: keep track of occupants, pressure points, and patient movement. Instantaneous data updates and modifications are possible with 6G connectivity, enhancing patient comfort and averting bedsores. Security features guarantee safety and patient privacy.
- Glucose monitors: check diabetes patients' blood sugar levels on a regular basis.6G ensures rapid data transfer to healthcare providers, enabling prompt interventions and modifications to treatment plans. Secure connections protect patient health data from unwanted access.
- Linked imaging systems: these comprise CT, MRI, and X-ray equipment that sends pictures for remote processing. 6G transfers large image files quickly, facilitating faster consultations and diagnoses. Security measures protect private medical images.
- Environmental sensors: monitor the lighting, temperature, humidity, and air quality in patient rooms and other crucial areas. 6G enables real-time control and monitoring, ensuring the best possible environmental conditions for patient safety and wellbeing. Improved security features prevent sensor data manipulation.
- Infusion pumps sensors: give patients precisely the right dose of medication. 6G connectivity guarantees rapid and precise data on medicine delivery, allowing for remote monitoring and modifications. Secure communication prevents potential errors or interference.
- Fall detection sensors: these devices identify falls in patients and sound an alarm. 6G's rapid data transfer speeds guarantee prompt notifications to medical professionals, cutting down on reaction times and enhancing patient security. Security mechanisms safeguard data about patient movements and locations.
- Telemedicine tools sensors: enable online consultations and exams. 6G raises the standard of telehealth services by providing the enormous bandwidth required for high-definition audio and video. Secure connections guarantee the confidentiality of patient-doctor communications.

Certain sensors require 6G security and quick speed features (62).

- Wearable health monitors and glucose monitors are essential for ongoing patient care; they need to transmit data in real-time and with a high level of security to safeguard private medical data.
- Connected imaging systems require strong security to protect private diagnostic images, as well as fast transmission speeds for large files.
- Infusion pumps require secure, instantaneous communication to ensure precise drug administration and prevent errors or manipulation.

#### 5.1.2 Key components level implementation of IoT in smart hospital

**Figure 4:**
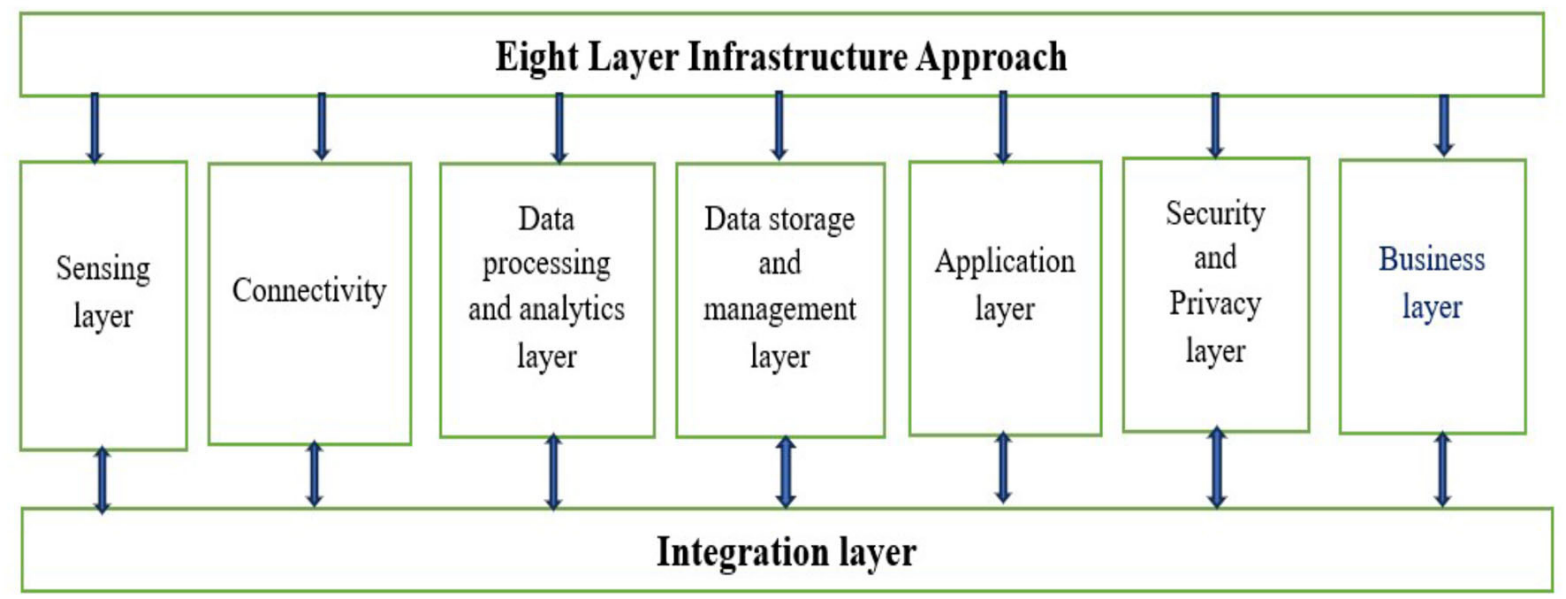
IoT architecture.

The architecture of an IoT-based smart hospital which was referenced in Philips Health Suite and Siemens IoT-enabled solutions for healthcare comprises several interconnected components and layers that enable seamless communication, data exchange, and intelligent decision making, as shown in Figure 4.

##### 5.1.2.1 Philips Health Suite Digital Platform architecture layer

The Philips Health Suite Digital Platform, an innovative healthcare platform, integrates data from various sources, including electronic health records (EHRs), IoT devices, and other healthcare systems. The Philips Health Suite Digital Platform's architecture streamlines the collection, integration, and evaluation of medical data, enabling more personalized patient care and enhanced operational efficiency in healthcare facilities. Healthcare businesses to use data to deliver tailored care, improve clinical outcomes, and improve patient experiences thanks to the architecture of the Philips Health Suite Digital Platform, which aims to build a single ecosystem (63). At its core, the Health Suite Digital Platform consists of several key components (64):

- The data ingestion layer: this layer is in charge of gathering data from many sources, including wearables, medical sensors, IoT devices, and EHR systems. It guarantees that information is entered into the platform safely and processed further.
- Data management and storage layer: after being gathered, the data is kept in a scalable and safe cloud-based storage system. This layer contains databases and data lakes that effectively handle and organize the enormous volumes of healthcare data.
- Data analytics and insights layer: to extract useful insights from the gathered data, the platform uses machine learning algorithms and sophisticated analytics. Personalized care interventions and predictive analytics are made possible by this layer, which analyses patient data to find trends, patterns, and possible health hazards.
- Application services layer: to facilitate the development of healthcare applications and services, the Health Suite Digital Platform offers a collection of application services and APIs (Application Programming Interfaces). These services make it easier to create custom healthcare solutions, integrate with third-party systems, and ensure interoperability amongst healthcare equipment.
- Security and compliance layer: the platform has strong security mechanisms in place to safeguard patient data and guarantee adherence to healthcare laws like HIPAA (Health Insurance Portability and Accountability Act). Security is of the utmost importance in the healthcare industry. To protect sensitive medical data, this layer has audit trails, access control methods, and encryption.

##### 5.1.2.2 Siemens IoT-enabled solutions for healthcare layers

Siemens' Digital Enterprise portfolio includes IoT-enabled healthcare solutions that offer a holistic architecture that combines edge computing, sensors, networking, data analytics, and security measures. Siemens wants to use these technologies to propel the digital transformation of healthcare, making patient-centered, cost-effective, and intelligent healthcare delivery possible (65). A s part of its Digital Enterprise portfolio, Siemens provides IoT-enabled healthcare solutions that are intended to streamline hospital operations, improve patient experiences, and improve clinical outcomes. Siemens' IoT-enabled healthcare solutions are built with a number of essential parts and tiers, all of which are necessary to provide integrated, data-driven healthcare services (66).

- Sensors and medical devices: a variety of sensors and medical devices placed throughout the hospital setting form the basis of Siemens' IoT-enabled healthcare solutions. These gadgets include sensors for facility management, imaging systems, lab apparatus, and patient monitors. These sensors gather numerous pieces of information about patient health, operational effectiveness, and environmental factors.
- Siemens' architecture incorporates a robust networking infrastructure to facilitate seamless communication between sensors, devices, and backend systems. Both wired and wireless networks are part of this infrastructure, which guarantees dependable data transfer and instantaneous connectivity. Siemens facilitates compatibility and integration with current hospital IT systems.
- Edge computing and data processing: Siemens uses edge computing skills to manage the enormous amount of data produced by sensors and medical equipment. Within the hospital's walls, edge devices preprocess and analyze data locally, cutting down on latency and bandwidth needs. This distributed computing architecture facilitates real-time monitoring and alerting for key events, as well as quick decision-making.
- Cloud platform and data analytics: to extract useful insights from healthcare data, Siemens' Digital Enterprise portfolio makes use of cloud computing and sophisticated data analytics. Siemens securely transfers sensitive and device data to cloud-based platforms for further processing. Siemens provides healthcare professionals with predictive analytics and decision support tools using AI and machine learning algorithms to identify important patterns, trends, and correlations in healthcare data.
- Integration with hospital systems: Siemens' Internet of Things (IoT)-enabled healthcare solutions easily integrate EHRs, hospital information systems (HIS), and other clinical applications. This guaranteeing data interchange and compatibility between various systems, this integration permits thorough patient care coordination and workflow optimization.
- Security and compliance: Siemens' IoT-enabled healthcare infrastructure places a high priority on security. Strong cybersecurity safeguards protect sensitive patient data and guarantee adherence to healthcare laws like GDPR and HIPAA. These measures include encryption, access restrictions, and threat detection. Siemens employs a multi-layered security strategy to reduce risks and defend against constantly changing cyberthreats.

#### 5.1.3 How 6G help to overcome the challenges of integrating IoT in smart hospitals

Although the integration of IoT in smart hospitals brings numerous benefits, it also presents several challenges that require careful management. Addressing these challenges necessitates a strategic approach, effective collaboration between IT and healthcare departments, robust governance frameworks, and the continuous monitoring and evaluation of IoT systems. By effectively navigating these challenges, hospitals can fully leverage the transformative potential of the IoT to enhance efficiency and patient-centricity in healthcare services. The following are some key challenges associated with integrating the IoT in a smart hospital (67).

- Interoperability: due to the fact that different companies manufacture many IoT devices and use different communication protocols, interoperability issues arise. It can be difficult to integrate many devices into a coherent system; this may call for specialized integration work.
- Security and privacy: because IoT devices frequently gather private medical information, hackers find them to be appealing targets. IoT security flaws might make patient data vulnerable to illegal access or jeopardize the reliability of medical systems.
- IoT devices need network connectivity in order to send and receive orders, which contributes to their reliability and resilience. Network outages or disturbances can impact the dependability of IoT-based systems, potentially impacting patient safety and care.
- Scalability: as hospitals install more IoT devices, managing and scaling the infrastructure to meet demand will become more challenging. Scalable solutions that maintain performance and dependability over a large number of devices are required by hospitals.
- Data management and analytics: we must efficiently gather, save, and examine the massive volumes of data generated by IoT devices. To extract useful insights from data created by the Internet of Things, hospitals need to have a strong data management and analytics infrastructure in place.
- Healthcare regulations, such as HIPAA in the United States, impose strict guidelines for safeguarding patient privacy and data security. Hospitals must make sure that their IoT-based systems comply with regulatory requirements in order to prevent negative legal and financial repercussions.

6G technology has the potential to resolve several of these issues (68):

- Improved connectivity: in comparison to earlier generations, 6G networks offer substantially faster data speeds, reduced latency, and more device density. This enhanced connectivity may support more IoT devices and enable real-time data transfer for vital uses like telemedicine and remote patient monitoring.
- Enhanced security: we anticipate 6G networks to have cutting-edge security features like improved authentication procedures and encryption algorithms to fend off cyberattacks and illegal access. Furthermore, 6G networks may use AI-driven security solutions to instantly identify and neutralize threats.
- 6G networks could enable edge computing capabilities, enabling the processing and analysis of data closer to its source. By processing sensitive data locally instead of sending it over the network, edge computing can lower latency, ease network congestion, and improve data privacy.
- AI-driven optimization: by utilizing AI algorithms, 6G networks are able to detect network outages, optimize network resources, and dynamically distribute bandwidth according to application demands. This AI-driven optimization may help IoT-based smart healthcare systems become more resilient and dependable.
- Regulatory compliance: 6G networks may use features like integrated encryption and data anonymization methods to help with regulatory compliance. These elements can help hospitals comply with regulations regarding patient data protection and privacy.
- By utilizing 6G technology, hospitals can overcome many of the obstacles associated with IoT-based smart hospital deployments, ultimately improving patient care results, and operational efficiency.

### 5.2 Explainable artificial intelligence

Explainable AI, or XAI, is the development of artificial intelligence systems that not only make precise forecasts or suggestions but also transparently explain their judgments and actions in the context of smart healthcare. In the medical field, where choices have a direct effect on patients' lives, XAI is essential for fostering a sense of confidence, enhancing communication between AI systems and medical personnel, and guaranteeing patient safety. XAI makes AI-driven healthcare solutions more interpretable and accountable by offering clear justifications for diagnosis choices, treatment strategies, and prognostic predictions. Because of this transparency, doctors can verify AI recommendations, comprehend the underlying logic, and apply their domain expertise to the decision-making process. By enabling doctors to prioritize patient safety and wellbeing while making better-informed and confident judgments, XAI ultimately promotes increased acceptance and implementation of AI technology in healthcare. Real-time processing of AI in 6G hospitals demands strong hardware infrastructure that can support enormous volumes of data at ultra-low latency. Advanced edge computing devices, AI accelerators such as GPUs, TPUs, and neuromorphic processors emulating brain-like efficiency to make quick and precise decisions are needed. Edge computing is also essential in reducing data transfer delays through data processing close to the source, for instance, in ICU monitoring or robotic surgeries. Moreover, ultra-reliable low-latency communication (URLLC) modules and high-frequency 6G antennas are required to ensure smooth connectivity over hospital networks (69). Power consumption is also a key issue since real-time AI applications such as predictive diagnostics and robotic surgical systems demand constant data analysis. Energy-efficient hardware, dynamic power management methods such as adaptive voltage and frequency scaling (DVFS), and smart workload allocation can help minimize energy consumption. Blending renewable energy sources, like solar power, and using AI-powered algorithms to manage and minimize energy usage are key to sustainability. Hospitals must embrace green computing principles and work with equipment vendors to develop hardware specific to healthcare AI workloads. Regulatory agencies must also create standards to guarantee energy-efficient deployment while ensuring system performance and reliability. In the end, both high-performance processing and energy efficiency will be required to make the next generation of intelligent, 6G-driven healthcare services possible (70).

**Figure 5:**
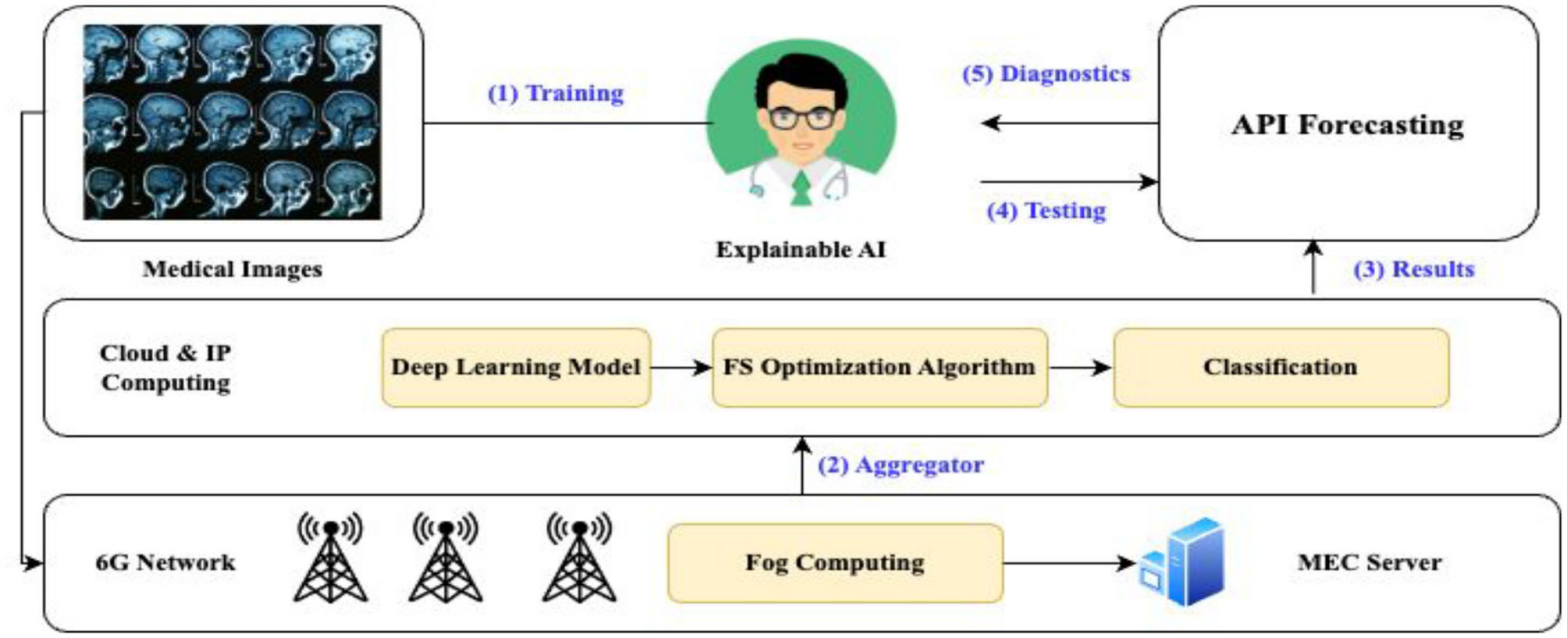
Self-explainable AI based smart healthcare.

An AI-based smart healthcare architecture that is self-explanatory incorporates AI algorithms that not only generate precise forecasts or suggestions, but also offer transparent, easily comprehensible explanations for their choices as shown in Figure 5. Interpretable AI models and methods that emphasize explainability over performance are the foundation of this architecture. This architecture uses AI algorithms to analyze healthcare data and produce predictions or recommendations. Examples of these algorithms include decision trees, rule-based systems, and interpretable deep learning models. These algorithms focus on producing precise results and providing clear justifications for their choices, highlighting the crucial elements or characteristics that influence the outcome. Additionally, the architecture has parts for showing patients and healthcare professionals AI-generated explanations. This could entail the use of interactive dashboards, graphical displays, or plain language explanations that make the logic underlying AI predictions simple to comprehend. Moreover, the design includes components for tracking and assessing AI model performance and interpretability over time. This guarantees that the AI system will always be trustworthy, transparent, and sensitive to the requirements and expectations of its users. By offering comprehensible justifications for AI-driven decisions, self-explanatory AI-based smart healthcare architecture promotes trust, accountability, and cooperation between AI systems and human stakeholders. This improves clinical decision-making, patient engagement, and overall healthcare outcomes.

#### 5.2.1 Types of data use by XAI

In the healthcare industry, explainable AI (XAI) uses a variety of medical data sources to offer clear and comprehensible insights into AI-driven decision-making procedures (71). These data sources include (72, 73):

- EHR: these records contain a patient's medical history, diagnosis, prescriptions, test results, and treatment plans. In order to help doctors make well-informed decisions about patient care, XAI algorithms examine EHR data.
- Medical imaging: data from modalities such as CT scans, MRIs, ultrasounds, and X-rays is processed using XAI algorithms. In order to help radiologists identify anomalies, make diagnoses, and schedule treatments, AI systems analyze imaging data.
- Genomic data: DNA sequences, gene expression profiles, and genetic variants are among the genomic data that XAI is used to interpret. AI systems examine genetic data to find genetic markers linked to specific illnesses, customize therapeutic strategies, and estimate the likelihood of developing a disease.
- IoT with wearable devices: XAI algorithms examine information gathered from wearable sensors and IoT devices that track physiological characteristics like as activity levels and vital signs. This information is used to monitor patient health, identify abnormalities, and offer early warning indicators of possible medical problems.

**Several instances of AI/ML algorithms in use in hospitals exist:**

- Deep learning in medical imaging: in medical imaging, convolutional neural networks, or CNNs, are frequently employed for tasks like disease categorization, lesion detection, and picture segmentation. For example, CNNs are used in the FDA-approved AI program IDx-DR to evaluate retinal pictures for the purpose of screening for diabetic retinopathy.
- Clinical decision support systems: clinical decision support systems are created using machine learning techniques like decision trees and random forests as well as rule-based systems. By evaluating patient data and medical literature, IBM Watson for Oncology, for instance, applies machine learning algorithms to help oncologists make therapy decisions.
- Natural language processing (NLP): NLP methods are used to extract structured data from narratives and unstructured clinical notes included in electronic health records (EHRs). NLP is used by Google's DeepMind Health to evaluate EHR data for purposes including treatment suggestions and patient risk assessment.

#### 5.2.2 AI/ML used in hospitals

The availability of large-scale medical datasets, advances in AI/ML methodologies, and increases in processing capacity, the use of such AI approaches in healthcare is still relatively new. These strategies are being actively used by healthcare organizations, academic institutions, and digital companies to enhance patient care, streamline clinical processes, and quicken medical research. Worldwide, a number of healthcare facilities and hospitals are utilizing diverse AI and machine learning (ML) algorithms to examine medical data for a variety of purposes. Here are a few instances (25, 74):

- United States' Mayo Clinic: to identify patients who may experience specific medical illnesses or complications, Mayo Clinic uses artificial intelligence (AI) and machine learning (ML) algorithms for predictive analytics. Additionally, they use natural language processing (NLP) algorithms to glean insights from electronic health records (EHR) and unstructured clinical notes.
- University College London Hospitals (UCLH) in the United Kingdom: UCLH uses artificial intelligence (AI) algorithms for medical imaging, radiology, and pathology image processing. These algorithms help evaluate medical pictures, such as MRIs, CT scans, and X-rays, so that doctors can diagnose illnesses and ailments more quickly and accurately.
- Seoul National University Bundang Hospital (South Korea): this hospital uses artificial intelligence (AI) to provide personalized care by evaluating genetic information and medical records to create customized treatment regimens and forecast patient reactions to various drugs and treatments.
- Massachusetts General Hospital (United States): based on past data and present health state, Mass General uses AI algorithms for clinical decision support, helping physicians diagnose illnesses, choose the best course of therapy, and forecast patient outcomes.
- Singapore General Hospital (Singapore): in order to improve the caliber and accessibility of healthcare services, this hospital uses AI and ML algorithms to manage healthcare operations. These algorithms optimize resource allocation, patient scheduling, and workflow efficiency.

#### 5.2.3 Benefit of XAI in 6G based smart hospital over 5G

Compared to 5G technology, the integration of XAI in a 6G-based smart hospital offers the following advantages (75):

- In a 6G smart hospital, XAI provides clear justifications for AI-generated suggestions and judgements. Healthcare workers must be able to comprehend the reasoning behind AI-generated insights in order to build trust in the technology and enable cooperation between AI systems and human clinicians.
- 6G-based XAI algorithms provide better interpretability when compared to AI models in 5G environments. As a result, physicians will be better equipped to verify suggestions and more successfully apply their domain knowledge to decision-making processes, as they will have a deeper understanding of how AI makes its decisions.
- XAI in a 6G smart hospital allows medical professionals to go back and confirm the logic behind particular suggestions or actions, which increases AI systems' accountability. This accountability is crucial for ensuring that AI-driven interventions comply with ethical and clinical criteria in healthcare settings where decisions have a direct influence on patient lives.
- XAI in a 6G smart hospital helps reduce the possibility of biases or mistakes in AI-driven decision-making by offering clear and understandable answers. Physicians can more readily recognize possible flaws or restrictions in AI systems, enabling them to step in when needed to protect patient safety and wellbeing.
- The use of artificial intelligence (AI) in healthcare can help to support regulatory compliance standards. XAI capabilities in a 6G smart hospital can assist. Healthcare businesses can demonstrate compliance with regulatory norms and rules governing the use of AI technologies in clinical practice by utilizing features such as transparent explanations and interpretability.

#### 5.2.4 How XAI can be integrated with 6G based smart hospital

Incorporating transparency and interpretability elements into AI-driven healthcare systems is necessary to integrate XAI with a 6G-based smart hospital. Healthcare companies can create AI-driven healthcare solutions that are more transparent, understandable, and reliable by combining XAI with 6G-based smart hospital systems. In the end, this improves patient outcomes by fostering human-machine collaboration and bolstering clinicians' faith in AI technologies. Here's how 6G technology can integrate XAI into a smart hospital setting (76):

- Algorithm design: create AI algorithms with interpretability and transparency as top priorities. This entails producing justifications for AI predictions or suggestions using methods like decision trees, rule-based systems, and model-agnostic methodologies.
- Real-time explanation generation: when AI algorithms make judgments or forecasts, implement systems that instantly produce explanations. Delivering these explanations in a format suitable for the current healthcare activity should enable healthcare workers to understand them.
- Integrating with 6G connectivity: make use of 6G networks' fast, low-latency connectivity to enable smooth communication between AI systems and healthcare organizations. Ensure that clinicians' devices can swiftly and reliably receive and process XAI explanations, enabling immediate review.
- User interface design: create user interfaces that display AI suggestions or forecasts, along with XAI explanations. This keeps their workflow uninterrupted and makes it simple for physicians to access and understand the logic underlying AI-driven decisions.
- Feedback mechanisms: put in place systems that let medical professionals comment on how relevant and accurate XAI explanations are. This gradually enhances the transparency and interpretability of AI systems by utilizing human judgment and input.
- Security and privacy: ensure the secure transmission of XAI explanations via 6G networks to protect patient confidentiality and privacy. To safeguard sensitive medical data during transmission, employ authentication and encryption techniques.
- Regulatory compliance: verify that the XAI integration conforms with the laws and regulations, including HIPAA and GDPR, that control the use of AI in healthcare. This requires transparency in the XAI explanation process and adherence to the accuracy and dependability standards established by regulations.

#### 5.2.5 Challenges of XAI and how 6G can help

In smart hospitals, XAI presents problems primarily related to accountability, transparency, and trust in AI-driven decision-making. 6G technology can help XAI in smart hospitals by facilitating clear communication, reducing prejudice, boosting security and privacy, and strengthening the resilience and dependability of AI-driven healthcare systems. Smart hospitals may implement XAI solutions that empower physicians, enhance patient outcomes, and promote confidence in AI-enabled healthcare delivery (77). Table 4 indicate some of the issues and possible solutions that 6G technology may bring about:

**Table 4:** Challenges and solution of XAI by 6G.

**Parameters**	**Challenges**	**How 6G can help**
Interpretability	One issue with AI in healthcare is that some algorithms are “black boxes,” making it challenging to figure out how they arrive at particular conclusions. In healthcare settings, where doctors must trust and comprehend the reasoning behind AI-driven suggestions, this lack of interpretability can be a challenge.	6G networks facilitate real-time communication between AI systems and healthcare providers, enabling the exchange of comprehensive justifications for AI-generated suggestions. 6G-enabled augmented reality (AR) devices, for instance, might instantly superimpose explanations onto patient data or medical images, giving medical professionals a clear visual representation of AI reasoning.
Fairness and bias	AI systems trained on inadequate or biased data may unintentionally exacerbate or prolong existing inequalities in healthcare outcomes. Ensuring justice and fairness in AI decision-making is essential to giving every patient access to high-quality care.	How 6G can help: 6G can facilitate the transfer of massive datasets required for training AI models on a variety of representative data sources due to its high bandwidth and low latency. Furthermore, the federated learning capabilities of 6G networks allow several institutions to cooperatively build AI models without exchanging private patient data, thereby reducing the risk of bias and promoting justice.
Security and privacy:	Health information is extremely private and governed by stringent laws, such as the Health Insurance Portability and Accountability Act (HIPAA) in the US. When implementing AI systems in smart hospitals, data security and patient privacy protection are top priorities.	To safeguard data transferred between IoT devices, AI systems, and cloud servers, 6G networks include cutting-edge encryption techniques and improved security features. Hospitals can use differential privacy and secure multi-party computation over 6G networks to analyze sensitive patient data while maintaining patient privacy and regulatory compliance.
Robustness and reliability	To guarantee patient safety and care continuity, AI systems installed in smart hospitals need to be robust and resilient. Serious repercussions for patient outcomes could result from system malfunctions or inaccurate AI forecasts.	How can 6G be useful? 6G networks' ultra-reliable low-latency communication (URLLC) capabilities, which offer low latency and high dependability, enable mission-critical applications such as remote patient monitoring, telemedicine, and surgical robotics. By lowering the number of single points of failure and processing data closer to the point of collection, redundant 6G network designs and edge computing resources can significantly improve the resilience of AI systems

The use of 6G in healthcare creates substantial regulatory loopholes because of the unprecedented speed, connectivity, and volume of data. Existing healthcare data protection regimes, like HIPAA in the United States and GDPR in the European Union, can be inadequate to deal with the intricacies of 6G networks, particularly in terms of real-time processing of data, cross-border data transfers, and AI-based medical decisions. To fill these loopholes, a harmonized, worldwide regulatory regime is needed. This structure should create uniform protocols for data sharing, encryption, and interoperability across borders while maintaining adherence to regional healthcare legislation. Homomorphic encryption, zero-trust architecture, and blockchain can be made mandatory to secure patient data. Regulatory authorities should also make real-time auditing mechanisms mandatory and demand transparent AI algorithms, making diagnostic decisions explainable and unbiased. Ethical issues around AI-powered diagnoses and robot-assisted surgeries need to be resolved by introducing guidelines focusing on patient safety, consent, and responsibility. Accurate legal liability for AI mistakes, complete clinician education, and integration of human review in key medical procedures are important. There needs to be an integration with AI developers, healthcare professionals, and ethicists with regulatory authorities to work together to set ethical standards for AI. Public education and patient awareness regarding AI participation in their treatment will also enhance trust. Finally, an evolving, open, and internationally harmonized legal framework is essential to provide secure, ethical, and compliant 6G-based healthcare systems.

AI-based decisions in a 6G-enabled hospital need to be strictly audited for fairness and bias to guarantee patient safety and fairness. Explainable and transparent AI models are essential, as they enable healthcare workers and regulators to see how decisions are reached. Auditing needs to involve periodic checks of AI algorithms, testing against varied datasets, and tracking for any indication of discriminatory results based on race, gender, or socioeconomic status. Ethical frameworks, including the application of fairness measures and the integration of human judgment in key decisions, are needed to reduce bias. In addition, external audits by independent regulatory agencies must be performed to ensure adherence to healthcare data protection regulations.

The heightened surveillance facilitated by 6G technologies in intelligent hospitals is of concern regarding privacy and consent. Ongoing monitoring of patients by IoT devices, facial recognition, and AI analysis may result in over-surveillance possibilities that compromise individual liberties and create vulnerabilities in data. Surveillance, though it enhances patient care through real-time action, has dangers of data loss and unauthorized entry. Tight regulatory systems and patient consent processes have to be in place to prevent abuse and make sure that the advantages of AI-based healthcare do not occur at the cost of patient privacy.

### 5.3 Robotics in 6G based smart hospital in robotics

**Figure 6:**
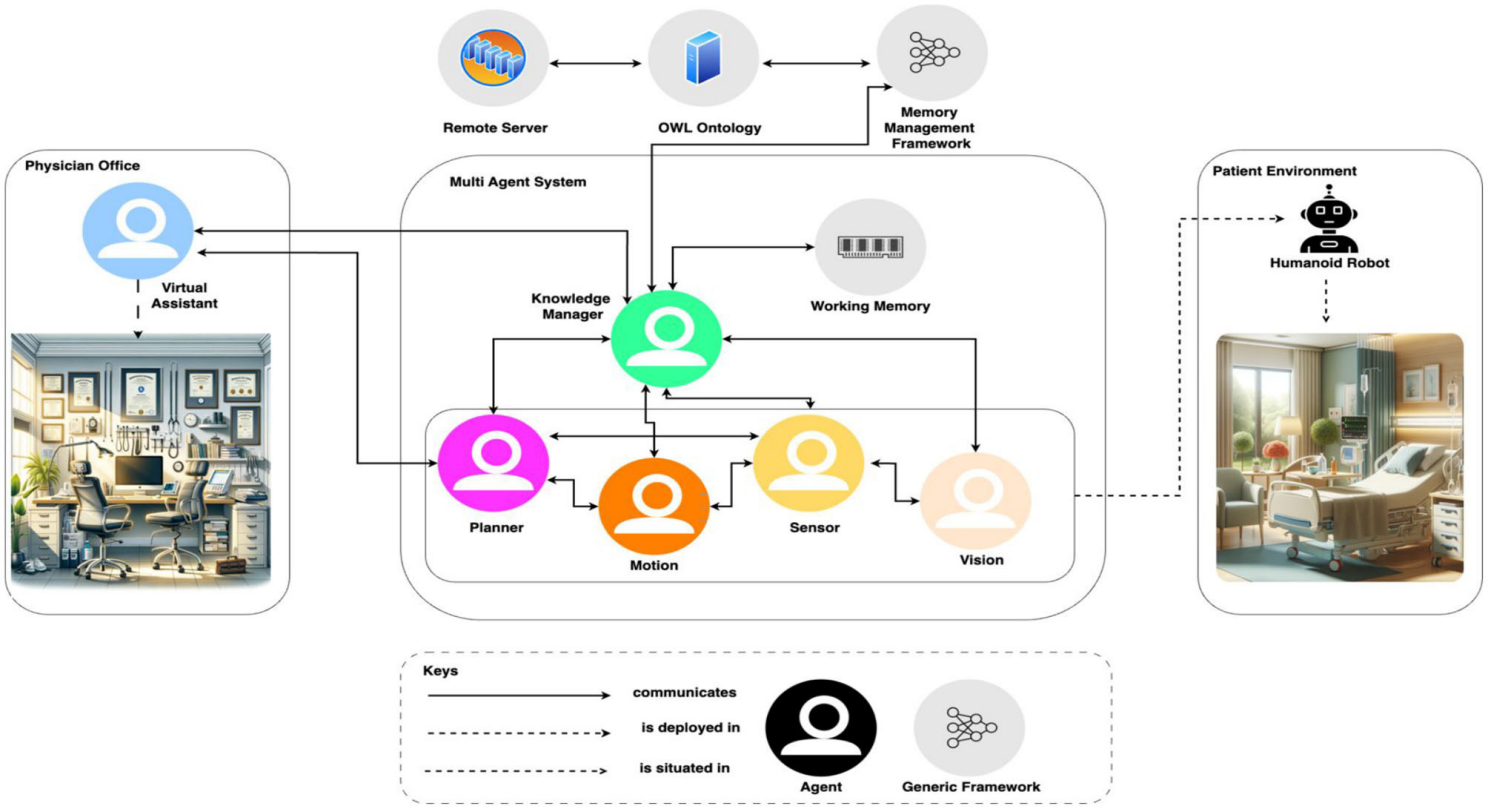
Robotic enabled smart health hospital.

6G-based smart hospitals will outperform their 5G counterparts and revolutionize robotics and automation by introducing innovative features. 6G networks' extremely low latency and large data rates make it possible to easily integrate sophisticated robotic systems, providing precise and real-time medical automation. The combination of ultralow latency, high data speeds, terahertz communication, and energy efficiency enhances robotics and automation in 6G-based smart hospitals. These characteristics enable a new wave of intelligent, flexible, and long-lasting robotic applications that will transform patient care and healthcare delivery (78). 6G's improved connectivity makes haptic feedback systems possible, which gives robotic treatment a tactile element. Ultra-low latency can help teleoperated robotic surgeries by allowing surgeons to accomplish complex tasks with previously unheard-of precision and responsiveness from a remote location. Healthcare settings can benefit from the adoption of swarm robots due to 6G's enhanced data speeds and better connection density. Swarm robots improve hospital operations by efficiently completing activities such as drug administration, sample collection, and environmental monitoring, while operating both cooperatively and independently. The combination of 6G with cutting-edge AI has enabled the development of more intelligent and context-aware robotic systems. These AI-powered robots can smoothly communicate with patients and medical personnel, adapt to changing hospital conditions, and move through congested areas with intelligence (79). Advanced medical imaging robots can perform high-resolution, real-time diagnoses because to 6G's terahertz communication capabilities. The AI algorithms of these robots enable them to examine medical images instantly, facilitating prompt decision-making and intervention. 6G focuses on sustainability and facilitates energy-efficient computing and communications. By prolonging robotic systems' operating life, lowering energy usage, and encouraging environmentally friendly automation techniques inside smart hospitals, this feature improves robotic systems. 6G enables more organic and cooperative interactions between people and machines. Healthcare workflows can easily include robotic assistants, sometimes known as humanoid robots, to support various duties such as patient care, rehabilitation exercises, and standard medical procedures (80). The robot-enabled smart hospital is shown in Figure 6.

#### 5.3.1 Advanced applications of Robots in smart hospital with statistics

Robotic surgery is a well-established technique; thus, robotic technology in hospitals is not new, especially when it comes to surgical procedures. However, outside of surgical settings, robotics technologies continue to bring new breakthroughs and applications to smart hospitals. The following is a list of recent and upcoming uses for robots in hospitals (81, 82):

- Robotic surgery: although already common, continuous improvements in robotic systems improve accuracy, adaptability, and efficiency, resulting in shorter recovery periods, fewer complications, and better patient outcomes. Studies have shown that the da Vinci Surgical System, for example, reduces blood loss and shortens hospital stays throughout a variety of minimally invasive procedures, such as hysterectomies, prostatectomies, and cardiac surgeries.
- Telepresence robots: these devices allow medical professionals to communicate with patients and provide care from a distance, facilitating virtual consultations and remote patient monitoring. In critical care situations or for patients with limited mobility, these robots let healthcare providers and patients communicate more easily.
- Logistics and distribution Hospitals are increasingly using autonomous robots for supply chain management, medicine distribution, and specimen transportation. These robots improve resource allocation efficiency, decrease manual work, and streamline hospital operations.
- Disinfection robots: due to the growing emphasis on infection control and hygiene, healthcare institutions use UV-C disinfection robots to sterilize patient rooms, operating rooms, and other high-touch surfaces. By lowering the risk of infections linked to healthcare, these robots enhance patient safety in general.

Even though robotic surgery is still a common use, there is more and more potential to integrate robots into hospital operations, improving patient care, efficiency, and infection control. The following data illustrates how robotic technology is affecting hospitals (83, 84):

- Studies have shown that using UV-C disinfection robots can reduce hospital-acquired illnesses by up to 50%.
- Robotic surgery has been associated with shorter hospital stays; in fact, some treatments have demonstrated a 40% reduction in stay time when compared to traditional surgery.
- Telepresence robots can increase patient satisfaction ratings by up to 25% by facilitating better access to care and communication between patients and healthcare professionals.

**Figure 7:**
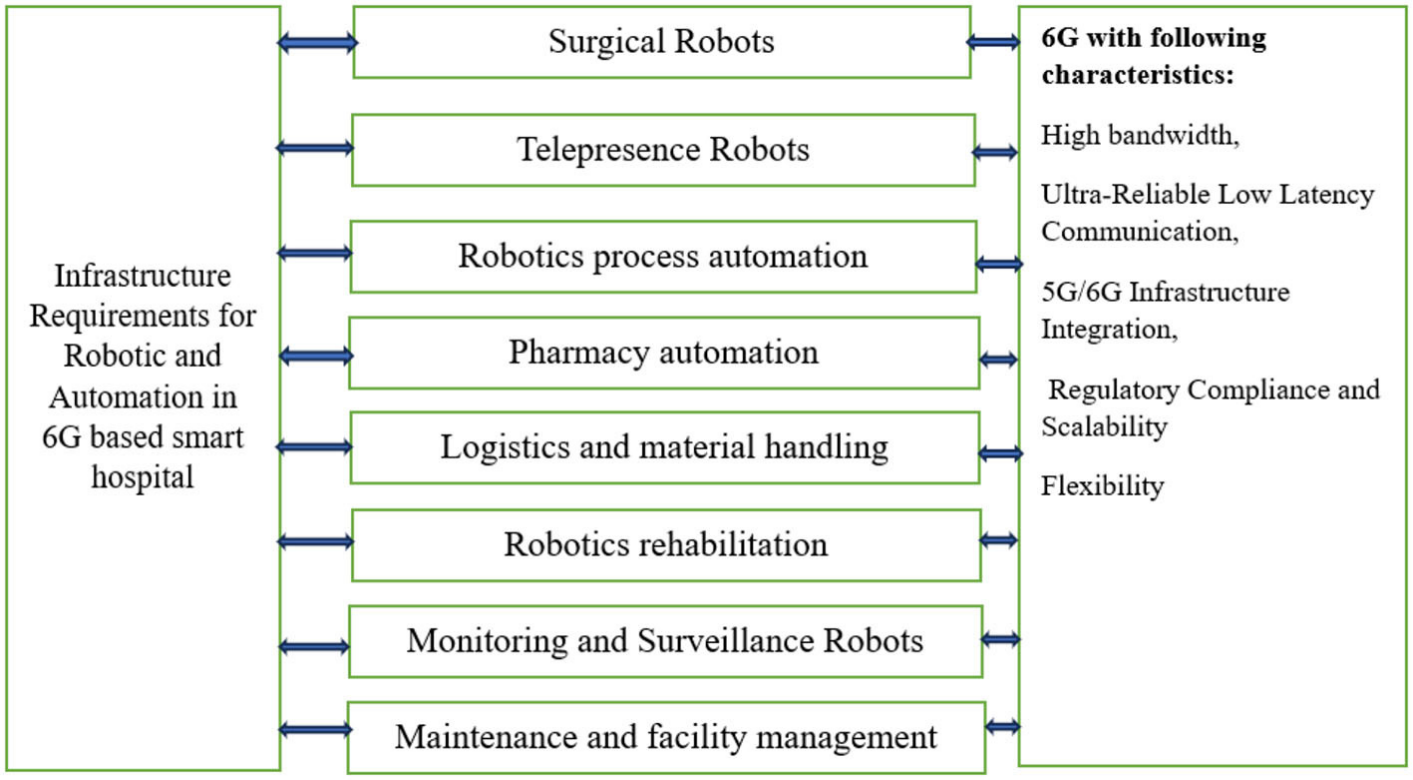
Infrastructure requirements for robotics and automation in 6G smart hospital.

Integrating robotics and automation into a 6G-based smart hospital requires meticulous planning, infrastructure preparedness, and attention to safety and regulatory requirements. Successful implementation hinges on collaboration between healthcare providers, technology vendors, and robotics specialists. Leveraging the synergy between robotics, automation, and 6G technology can significantly enhance efficiency, accuracy, and patient outcomes in healthcare environments. The architectural requirements are shown in Figure 7. Robotics and automation can be integrated in several ways (85, 86).

- **Surgical robots**: surgical robots enhance the precision and control of minimally invasive procedures. By integrating these robots with 6G networks, real-time communication and collaboration between surgeons and robots can be achieved. This allows surgeons to remotely control robots, execute complex procedures with increased dexterity, and utilize haptic feedback to improve surgical outcomes.
- **Telepresence robots**: telepresence robots are equipped with cameras, displays, and sensors to facilitate remote patient monitoring and virtual consultations. These factors allow health care professionals to interact with patients from afar. In a smart hospital utilizing 6G technology, telepresence robots take advantage of high bandwidth and low latency connectivity for real-time video communication, enabling healthcare professionals to assess patients remotely, provide guidance, and monitor their conditions effectively.
- **Robotic Process Automation (RPA):** Robotic Process Automation (RPA) automates repetitive and rule-based tasks in hospital workflows. In smart hospitals, RPA streamlines administrative processes such as patient registration, appointment scheduling, and billing. Automating these tasks helps reduce errors, enhance efficiency, and allow healthcare professionals to dedicate more time to patient care.
- **Pharmacy automation**: robotic systems in pharmacies automate medication dispensing, inventory management, and prescription filling. These systems handle medication orders with high accuracy and efficiency, reduce errors, and enhance medication safety. When integrated with 6G networks, these robotic systems enable real-time inventory tracking, automatic restocking, and seamless communication with healthcare providers to effectively manage medication.
- **Logistics and material handling:** robotics and automation play key roles in logistics and material handling within hospitals. Autonomous robots are deployed to navigate hospital premises, transport supplies, deliver medications, and assist with the movement of equipment and materials. When integrated with 6G networks, these robots achieve efficient task allocation, real-time tracking, and effective coordination, thereby enhancing the overall efficiency of hospital operations.
- **Robotic rehabilitation**: robotic systems are instrumental in patient rehabilitation and offer targeted exercises, support, and feedback to aid recovery. These systems are particularly beneficial for patients with mobility impairments, because they provide personalized therapy sessions and monitor progress. With the integration of 6G networks, these robotic systems allow for real-time monitoring, remote supervision, and personalized adjustments to therapy programs, thereby enhancing the efficacy of rehabilitation treatments.
- **Monitoring and surveillance robots**: robots equipped with sensors and cameras are used for monitoring and surveillance in hospitals. These robots can track vital signs, detect anomalies, and improve patient safety. The integration of these robots with 6G networks facilitates seamless data transmission, enabling real-time alerts and remote monitoring by healthcare professionals, thereby bolstering hospital security and patient care efficiency.
- **Maintenance and facility management**: robotics and automation play vital roles in hospital maintenance and facility management. Autonomous robots are deployed for routine inspection, equipment maintenance, and environmental monitoring. They efficiently identify and report issues, ensure prompt maintenance, and reduce equipment downtimes. With the integration of 6G networks, these robots facilitate efficient task management, support remote diagnostics, provide real-time status updates, and optimize hospital operations.

#### 5.3.2 Challenges and 6G solution in implementation of robotics in smart hospital

6G connectivity can overcome the implementation challenges of robotics in smart hospitals by providing the necessary infrastructure for real-time communication, remote operation, data processing, and security, ultimately enhancing patient care delivery and operational efficiency. Integrating robotics and automation in a 6G-based smart hospital presents several challenges that must be addressed. The following are some of the key challenges (87, 88):

- Integration complexity: it can be difficult to integrate robotic systems into the current hospital infrastructure, necessitating major adjustments to the physical layouts, operational procedures, and IT infrastructure.
- Safety concerns: when using robots in healthcare environments, safety must come first because mistakes or malfunctions could endanger patients or cause accidents. Ensuring regulatory compliance and a safe environment for robots to engage with patients are critical.
- Training and education: to effectively operate and interact with robotic devices, healthcare workers require specific training. It is necessary to create and conduct training programs to guarantee staff competence and assurance when utilizing robotic technologies.
- Costs and return on investment: for robotics systems, upfront investments in equipment, maintenance, and training are often significant. In contrast to conventional care delivery models, hospitals must evaluate the robotic solutions' long-term cost-effectiveness and return on investment (ROI).
- Interoperability: for smooth communication and data transmission, it is crucial to provide interoperability between various robotic platforms, medical equipment, and hospital IT systems. Interoperability and data integration require standardized interfaces and protocols. Patching legacy systems in current hospitals with 6G technologies will need a well-thought-out plan to facilitate seamless transition and interoperability. Legacy systems, including Electronic Health Records (EHR), imaging equipment, and older diagnostic equipment, commonly use old communication protocols and infrastructure. To fill the gap, hospitals will have to implement middleware solutions and software adapters that allow such systems to interact with newer 6G-compatible devices and applications. Furthermore, network updates like moving toward hybrid cloud-edge models will facilitate integrating existing sources of data with greater throughput and less latency that is offered by 6G. The process of integration will also include the upgrade of legacy hardware to accommodate 6G-compatible standards, including low-power IoT sensors and AI-based devices for real-time monitoring. Notably, this process must ensure data security and healthcare regulation compliance to safeguard patient privacy. By embracing scalable, modular solutions, hospitals can future-proof their infrastructure while ensuring compatibility with current systems.

6G connectivity can assist with these issues by performing the following tasks (89):

- Minimal latency and maximum bandwidth: 6G networks provide incredibly low latency and maximum bandwidth, allowing robotic system management and real-time communication. This guarantees that commands and actions happen as quickly as possible, improving the responsiveness and agility of robotic platforms.
- Support for edge computing: by enabling data processing and analysis closer to the source of data generation, 6G networks' edge computing capabilities lower latency and bandwidth consumption. This increases the autonomy and efficiency of robotic systems by enabling real-time decision-making and feedback loops.
- Remote operation and monitoring: surgeons and other healthcare professionals can remotely operate robotic devices for telemedicine and telesurgery applications because of 6G's high-speed, low-latency connectivity. This enhances patient care outcomes by providing access to medical services and specialist knowledge regardless of one's location.
- Security and reliability: to safeguard data sent between robotic equipment and hospital IT infrastructure, 6G networks include cutting-edge security features including encryption, authentication, and intrusion detection. This reduces the risks posed by cyberattacks and illegal access, improving the security and dependability of robotic operations.

### 5.4 Analyzing real problem in Thailand hospital and solving with 6G based smart hospital

The high maintenance costs of access points in Thailand's public hospitals negatively impact the quality and accessibility of healthcare, compounded by tight resources and posing numerous obstacles for the general population (90). Population hospitals may offer more dependable and effective services by utilizing cutting-edge technologies to reduce their high maintenance costs. This would immediately benefit the general population by improving their access to high-quality healthcare (91). The following are the effects this issue has on the broader public (16, 92):

- **Decreased quality of care**
  ***Equipment downtime*****:** longer downtimes resulting from medical equipment malfunctions frequently caused by poor maintenance can cut into the availability of crucial therapeutic and diagnostic services.
  ***Treatment delays***: individuals may encounter delays in the provision of medical care or diagnostic services, thereby exacerbating health effects, particularly in urgent or essential circumstances.
- **Extended waiting periods**
  ***Overburdened facilities***: when the remaining functioning equipment is out of commission, it leads to extended patient wait times. This is especially troublesome in high-demand fields like radiology and emergency departments.
  ***Appointment backlogs***: when maintenance problems cause a backlog of appointments, patients may have to wait longer for planned consultations and procedures, which may worsen their health.
- **Higher cost**
  ***High healthcare costs for patients***: if public hospitals are unable to provide prompt services, patients may be compelled to seek care from private hospitals, resulting in higher out-of-pocket costs.
  ***Indirect expenditures***: patients' overall healthcare expenditures may rise as a result of treatment delays that prolong sickness and necessitate more involved and costly therapies down the road.
- **Restricted availability of specialized services**
  ***Availability of specialized equipment***: regular maintenance is necessary for specialized diagnosis and treatment equipment, such as CT scanners and MRI machines. High maintenance expenses may restrict the provision of these treatments in public hospitals, thereby requiring patients to travel great distances to receive the necessary care.
  ***Equity issues***: health inequities between urban and rural populations may worsen in rural and underserved areas due to restricted access to specialist equipment.
- **Effect on hospital**
  ***Staff efficiency***: when dealing with broken or unavailable equipment, medical personnel may experience elevated stress levels and lower productivity, which may have an adverse effect on their capacity to deliver high-quality care.
  ***Instruction and adjustment***: frequent equipment failures and the introduction of temporary solutions can disrupt the workflow, forcing personnel to constantly adjust to changing circumstances and potentially impacting the overall performance of the hospital.
- **Public health consequences**
  ***Control of infectious diseases:*** to prevent the transmission of infectious diseases, it is essential to use dependable equipment and perform routine maintenance. Equipment malfunctions can jeopardize public health by impeding diagnostic capabilities and delaying the use of control measures.
  ***Handling chronic illnesses***: timely therapies and routine monitoring are essential for the effective management of chronic conditions such as hypertension and diabetes requires timely therapies and routine monitoring. Problems with equipment maintenance can interfere with continuing care strategies and worsen the health of individuals with chronic illnesses.

The above stated problem of high maintenance costs of access points in Thailand's public hospitals can be solved by deploying 6G networks and smart hospital technologies in the following manner:

- **Predictive upkeep:**
  Predicting equipment failures with IoT sensors and data analytics may guarantee prompt maintenance, cutting downtime, and preserving service availability.
- **Remote diagnosis:**
  High-speed, dependable remote diagnostics made possible by 6G networks allow professionals to handle maintenance issues without the need for in-person presence, resulting in faster problem resolution.
- **Optimized allocation of resources:**
  By making the most use of the resources at hand, smart systems can minimize service interruptions by prioritizing maintenance on vital equipment.
- **Enhanced effectiveness:**
  AI and automation can help hospitals run more efficiently, which will ease the workload for employees and increase the effectiveness of healthcare delivery as a whole.

The datasets used for experimental validation in the context of integrating 6G technology in smart hospitals should exhibit specific characteristics to accurately reflect real-world healthcare scenarios.

- Size: given the large-scale nature of smart hospitals, the datasets must be extensive, encompassing patient records, medical imaging, sensor data from IoMT devices, and real-time communication logs. These datasets should cover various aspects of healthcare, from diagnostics to treatment monitoring, to assess the impact of 6G-enabled solutions on data processing speed, latency, and bandwidth requirements.
- Diversity: the datasets should be diverse, representing a wide range of patient demographics, health conditions, and healthcare environments. This diversity is crucial to evaluating the performance of 6G in handling different medical applications, such as telemedicine, remote surgeries, and AI-driven diagnostics. The data should include structured formats (e.g., EHR) and unstructured formats (e.g., medical images, video feeds) to simulate the varied data inputs in smart hospitals.
- Challenges: one major challenge is ensuring data privacy and security, as sensitive patient information must be protected while transmitting over high-speed 6G networks. Additionally, data heterogeneity could pose integration issues, requiring effective data harmonization techniques. The computational complexity involved in handling large datasets for AI and real-time analytics also demands advanced processing capabilities, which could be another hurdle in the experimental setup.

### 5.5 Hybrid cloud-edge computing

Hybrid cloud-edge computing solutions present a strong alternative to 6G infrastructure in healthcare with an effective combination of cloud computing's scalability and edge computing's low-latency benefits. In healthcare, where real-time data processing and rapid decision-making are essential, this hybrid approach can maximize performance and cost-effectiveness. Edge computing devices situated near medical devices, including wearables, monitoring devices, and surgical robots, handle the processing of data locally, minimizing latency and the requirement for ongoing cloud communication. Local processing is essential in time-sensitive applications like remote surgery and real-time patient monitoring, where delays can be disastrous (93). Alternatively, cloud computing offers centralized storage, computational capacity, and support for high-volume data analysis, useful for predictive diagnostics, machine learning, and patient record keeping. Cloud resources are capable of managing the heavy computational workloads of AI algorithms without loading edge devices with processing large amounts of data, thus providing high-level analytics and data backups without loading local infrastructure (94). A hybrid architecture alleviates the weaknesses of both cloud and edge computing. It ensures that healthcare systems are not totally reliant on cloud infrastructure, which is costly or prone to outages, and also refrains from the performance constraints of edge computing. Hybrid systems can enhance scalability, flexibility, and reliability, particularly for remote locations with poor network connectivity, where edge devices can operate independently. By integrating these technologies, healthcare systems can deliver ongoing, real-time care, maximize resource utilization, and maintain strong data privacy and security through local processing and cloud storage (95, 96).

## 6 Conclusion

This projected article presents a comprehensive study of 6G-based smart hospitals, exploring the architectural evolution, advanced techniques, and challenges associated with this cutting-edge healthcare paradigm. Our research highlights the transformative potential of 6G technology in revolutionizing healthcare delivery. The architectural evolution emphasizes the seamless integration of diverse technologies to create a robust and interconnected healthcare ecosystem. Advanced techniques such as Explainable AI, IoT, and Robotics optimize patient care, resource management, and operational efficiency, enhancing diagnostic accuracy, streamlining workflows, and improving patient outcomes. However, our study also reveals significant challenges accompanying 6G implementation in smart hospitals, including security and privacy concerns, interoperability issues, and the need for substantial investments. Striking a balance between innovation and security is crucial for widespread adoption. This study provides a roadmap for researchers, practitioners, and policymakers to navigate the evolving landscape of 6G-based smart hospitals as we stand on the cusp of a new era in healthcare technology characterized by unprecedented connectivity and intelligence. Future work should focus on fortifying security and privacy, developing robust encryption methods, authentication protocols, and privacy-preserving mechanisms to mitigate risks and ensure data integrity. Research should also explore user experience, human-machine interaction, and the integration of patient feedback to create technologies that enhance healthcare delivery while prioritizing the wellbeing of patients and providers. Limitations include high implementation costs, data security concerns, the need for advanced infrastructure, and the lack of detailed analysis of ethical issues and potential disparities in technology access.

## Data Availability

The raw data supporting the conclusions of this article will be made available, upon reasonable request to the corresponding author.
